# Supplementary material for: Experiences and meanings of integration of TCAM (Traditional, Complementary and Alternative Medical) providers in three Indian states: results from a cross-sectional, qualitative implementation research study
Source: BMJ Open. 2014 Nov 25;4(11):e005203. doi: 10.1136/bmjopen-2014-005203 (PMC4248091; doi:10.1136/bmjopen-2014-005203)
Supplement: Author's manuscript [file bmjopen-2014-005203.draft_revisions.pdf]

BMJ Open

Experiences and meanings of integration of TCAM (Traditional, Complementary and Alternative Medical) providers in three Indian states: Results from a cross-sectional, qualitative implementation research study

Journal:	*BMJ Open*
Manuscript ID:	bmjopen-2014-005203
Article Type:	Research
Date Submitted by the Author:	07-Mar-2014
Complete List of Authors:	Nambiar, Devaki; Public Health Foundation of India,<br>Narayan, Venkatesh; All India Institute of Medical Sciences,<br>Lakshmi, JK; Indian Institute of Public Health - Hyderabad,<br>Porter, John; London School of Hygiene & Tropical Medicine, Department of Clinical Research<br>Sathyanarayana, TN; Indian Institute of Public Health-Bangalore Campus, Health Policy and Management<br>Sheikh, Kabir; Public Health Foundation of India,
**Primary Subject Heading**:	Health services research
Secondary Subject Heading:	Global health, Qualitative research
Keywords:	COMPLEMENTARY MEDICINE, International health services < HEALTH SERVICES ADMINISTRATION & MANAGEMENT, QUALITATIVE RESEARCH

SCHOLARONE™
Manuscripts

“Little somethings,” but “nothing as such”

Running Head: “Little somethings,” but “nothing as such”

Title: Experiences and meanings of integration of TCAM (Traditional, Complementary and Alternative Medical) providers in three Indian states: Results from a cross-sectional, qualitative implementation research study

Authors: D Nambiar,[1] VV Narayan,[2] JK Lakshmi,[3] J Porter,[4] TN Sathyanarayana,[5] K Sheikh[q]

1 Public Health Foundation of India, New Delhi, India

2 All India Institute of Medical Sciences, New Delhi, India

3 Indian Institute of Public Health, Hyderabad, India

4 London School of Hygiene and Tropical Medicine, London, United Kingdom

5 Indian Institute of Public Health, Bangalore, India

Corresponding Author Information:

Devaki Nambiar, PhD

Research Scientist

Public Health Foundation of India

ISID, 4 Institutional Area

Vasant Kunj 110067

Keywords: Alternative Medicine, Qualitative Evaluation, World Health

Word Count: 3,075

For peer review only

For peer review only - http://bmjopen.bmj.com/site/about/guidelines.xhtml

"Little somethings," but "nothing as such"

Abstract

Objectives: Efforts to engage Traditional Complementary and Alternative Medical (TCAM) practitioners in the public health workforce have growing relevance for India's path to universal health coverage. We used an action-centred framework to understand how policy prescriptions related to integration were being implemented in three distinct Indian states.

Setting: Health departments and district-level primary care facilities in the states of Kerala, Meghalaya, and Delhi.

Participants: In each state, two or three districts were chosen that represented variation in accessibility and distribution across TCAM providers (e.g.,small or large proportions of local health practitioners, homoeopaths, Ayurvedic and/or Unani practitioners). Per district, two blocks or geographical units were selected utilizing the criteria of proximity from district headquarters. TCAM Practitioners, administrators and representatives of community at district and state levels were chosen based on their putative roles indicated in publicly available records from state and municipal authorities. A total of 196 interviews were carried out: 74 in Kerala, and 61 each in Delhi and Meghalaya.

Primary and secondary outcome measures: We sought to understand experiences and meanings associated with integration across stakeholders, as well as barriers and facilitators to implementing policies related to integration of TCA providers at the systems level.

Results: We found that individual and interpersonal attributes tended to facilitate integration, while system features and processes tended to hinder it. Collegiality between individuals, stature and initiative of individual practitioners, along with high-level political will and/or individual access to top decision-makers enabled integration. The system was characterised, on the other

For peer review only - http://bmjopen.bmj.com/site/about/guidelines.xhtml

hand, by limited channels of formal communication across systems of medicine, inappropriate design of service delivery, particularly in co-located facilities, and fragmented administrative structures.

Conclusions: Strategies that attempt to make the health systems isomorphic or receptive to individual integrative efforts may facilitate integration across systems, creating opportunities for greater collaboration and trust.

Strengths

* Multi-sited qualitative study drawing on meanings and experiences across patients, providers, and health systems administrators

* Implementation research using rigourously applied interpretive policy analysis methods

* Linked to India's path on Universal Health Coverage

Limitations

* Cross-sectional study, so other than self-report of historical changes, we were not able to chart or map changed views or experiences of participants in vivo.

* Focus on the public service delivery sector, even as a great deal of health-seeking takes place in the private sector, with the assumption that public sector strengthening is highly desirable, and possible only through focused study on it

For peer review only - http://bmjopen.bmj.com/site/about/guidelines.xhtml

"Little somethings," but "nothing as such"

Introduction

The 1978 Alma Ata declaration called for traditional medicine, treatments and practices to be "preserved, promoted and communicated widely and appropriately based on the circumstances in each country." Thirty years later, the 2008 Beijing Declaration on Traditional Medicine called for integration of providers into national health systems, recommending systems of qualification, accreditation, regulation and communication (with allopathic providers).[1] These features of the Beijing Declaration were echoed at the 62nd World Health Assembly in 2009, putting out a call to action to United Nations member states to move forward with their plans for integration.[2] The global positioning of Traditional, Complementary, and Alternative Medicine (TCAM) has issued from and tends to imply a central focus on clinical and experimental medicine,[3] yet, recent calls for health systems integration,drawing attention to features like education, accreditation, regulation, and health services provision,place greater attention upon the TCAM health workforce.

In earlier work, we have identified three broad trends of integration as it relates to TCA providers: self-regulation with governmental linkage, government regulation and provisioning, and hybrid/parallel models.[4]This links roughly to the WHO nosology, where three models are identified: "tolerant" systems where the national health care system is based entirely on biomedicine but some TCAM practices are legally permissible, "inclusive" systems where TCAM is recognised but not fully integrated into all aspects of healthcare, and "integrative," where TCAM is officially recognised in national drug policy, providers and products are registered and regulated, therapies are widely available and covered under insurance schemes, research and education are widely accessible.[5]

The situation on the ground in India, hybrid in our view, seems in parts to reflect tendencies across WHO categories. The dominance of biomedicine appears to be a critical feature of

For peer review only - http://bmjopen.bmj.com/site/about/guidelines.xhtml

postcolonial health system, even as pre-independence, the TCAM practitioner community had played a major role in resisting colonial domination in the practice of (bio)medicine.[6] In part as a response to the reliance on allopathy throughout modern Indian history, there have been strong arguments in favour of the critical role that non-mainstream practitioners play in offering accessible, affordable, and socially acceptable health services to populations [1,7,8] . A study in Maharashtra reported that the situation of traditional healing as a community function through shared explanatory frameworks across provider and patient are explicitly unlike typical doctor-patient relationships.[7]

In India, one can also find a larger integrative framework, one that mandates "mainstreaming" of codified TCAM in India, collectively referred to as AYUSH, an acronym for Ayurveda, Yoga & Naturopathy, Unani, Siddha, Sowa-Rigpa, and Homeopathy. The National Rural Health Mission (NRHM), launched in 2005 to fortify public health in rural India, took a particular interest in integrating AYUSH practitioners through facilitation of specialised AYUSH practice, integration of AYUSH practitioners in national health programmes, integration of AYUSH modalities in primary health care, strengthening the governance of AYUSH practice, supporting AYUSH education, establishing laboratories and research facilities for AYUSH, and providing infrastructural support.[8] Human resource-focused strategies included contractual appointment of AYUSH doctors in Community and Primary Health Centres, appointment of paramedics, compounders, data assistants, and managers to support AYUSH practice, the establishment of specialised therapy centres for AYUSH providers; inclusion of AYUSH doctors in national disease control programmes; and incorporation of AYUSH drugs into community health workers' primary health care kits. A recent report from the AYUSH department reports that NRHM has established AYUSH facilities in co-location with health facilities in many Indian states (notably, not in Kerala, where the stand-alone AYUSH facility is the chosen norm). As of 2012, more than three quarters of India's district hospitals, over half of its Community Health

For peer review only - http://bmjopen.bmj.com/site/about/guidelines.xhtml

"Little somethings," but "nothing as such"

Centres and over a third of India's Primary Health Centres have AYUSH co-location, serving about 1.77 million, 3.3 million, and 100,000 rural Indians, respectively.[9]

And yet, even this integration framework has at most an "inclusive" character. This is reflected in findings like "official neglect" of traditional orthopaedic practitioners who have no registration, uniformity in inter-state regulation, or institutionalized medical training.[10]AYUSH doctors contracted to Medical Officer posts in Primary Health Centres (PHCs) in Andhra Pradesh report numerous lacunae in the implementation of the mainstreaming initiatives in the National Rural Health Mission (NRHM):[11] job prerequisites are not indicated, no benefits or allowances provided for health, housing or education, and compensation packages are much lower than those of allopathic doctors. Support for AYUSH practice is also inadequate (lack of infrastructure, trained assistants, and drug supply) and unethical practices have also been reported (documenting attendance of absentees, non-AYUSH personnel refusal to collaborate).Evidence from NRHM suggests that reshuffled AYUSH providers practice forms of medicine beyond the scope of their training.[12]Paradoxically, moreover, some Indian states prohibit cross-system prescription, adding ethical dilemmas for TCA practitioners who serve as the only medical practitioner in resource-poor areas.[26]

At a larger scale, current practices of integration (as in NRHM) have been described as substitution and replacement; which tend to ignore the merits of TCAM and present more barriers than facilitators of integration.**Error! Bookmark not defined.**Particularly given the strong push towards co-location and other strategies of integration as part of India's move towards Universal Health Coverage however, the integration of AYUSH practitioners could result in a doubling of the health workforce. And yet, there are strong fears that such an emphasis on quantitative aspects of integration, i.e. having the right number of practitioners placed at facilities is inadequate. There is a need to critically appraise the government infrastructure to support TCA, identify barriers and facilitators to integration that have emerged

For peer review only - http://bmjopen.bmj.com/site/about/guidelines.xhtml

"Little somethings," but "nothing as such"

from this rapid placement of these practitioners, and how these TCA practitioners, allopathic practitioners, and health system actors are reacting and adapting to each.

Methods

This analysis draws from a larger mixed methods implementation research study aimed at understanding operational and ethical challenges in integration of TCA providers for delivery of essential health services in three Indian states.

Our study was based on action-centred frameworks[13]with a focus on policy *actors* and *processes*.[14]We have therefore sought to understand the implementation of integration policies empirically. A team of four field researchers wasoriented by the principal investigator and advisor to the post-positivist paradigm of research, using Yanow's model of interpretative policy analysis, where the emphasis is equally on describing the experience of policy processes, and on elaborating the meanings actors attach to those processes.[15]The research protocol was approved by the Institutional Review Board of the Public Health Foundation of India.

Our methods included semi-structured in-depth interviews with policymakers, administrators, TCAM and allopathic practitioners, traditional healers, health workers and community representatives in three diverse Indian states: Kerala, where a number of systems have strong historical and systemic roots (N=74), Meghalaya, where local health traditions hold sway (N=61), and Delhi, where national, state, and municipal jurisdictions interface with multiple systems of medicine (N=61). Participants were selected based upon maximum variation criteria for each category. We sought to represent different schemes, levels of implementation (directorates, zonal officers), systems of medicine, types of establishments (hospital, dispensary), and years of experience.

In each state, one senior researcher, a research associate and a field researcher developed selection matrices to achieve maximum variation across each category of respondents. In each

For peer review only - http://bmjopen.bmj.com/site/about/guidelines.xhtml

state, two districts were chosen (in the case of Delhi, three municipal zones) that represented variation in accessibility and distribution across TCA providers (eg. small or large proportions of local health practitioners, homeopaths, Ayurvedic and/or Unani practitioners). Publicly available records from state and municipal authorities were consulted in order to determine location and type of facility (co-located, stand-alone) as well as suggestions and recommendations from Key Informants. Interviews were undertaken only with prior informed consent, and separate consent to record interviews. Data were were transcribed and stored in password-protected folders and each transcript was checked by investigators for corrections and quality of transcription.

Textual data from transcripts of interviews were analysed through a combination of deductive and inductive techniques in the "framework" approach of qualitative analysis for applied policy research[16]using ATLAS.ti7 software. Themes were developed in three iterations: in the first stage, the lead researcher from each state applied *a priori* codes and closely perused transcripts to devise *emergent* codes, with the support of the research associate. Researchers coded part of each other's state datasets to ensure that codes were being applied in a similar, uniform manner. In the second stage, agreement and consolidation of emergent codes across three sites took place under the direction of the study lead; these were then applied to data from each state by its respective lead researcher. Concurrently, lead researchers developed super-codes, or *analytic* codes to group emergent codes. The study lead finalised and then indexed across sites to arrive at results. Emergent and analytic code families were used to develop analyses, involving sharing of data and consultation across sites. We present emergent codes related to experiences and interpretations of integration.

Results

Individual experiences and meanings – collaboration and trust

For peer review only - http://bmjopen.bmj.com/site/about/guidelines.xhtml

"Little somethings," but "nothing as such"

We found that collaboration and trust appeared to be features of individual and interpersonal relationships across providers and system actors. For instance, many reported collegiality between and across TCA and allopathic practitioners. In Meghalaya, an allopathic medical officer noted that in some places Ayurvedic and homeopathic doctors were collaborating closely with his colleagues, expressing an interest in learning more about allopathic practices.

Another aspect was the "stature" of individual practitioners In Kerala,an Ayurvedic practitioner noted that: "Nobody can question MSV if he says that taking *chavanaprasham*[health paste] will lead to DNA repair, then nobody can question because they are saying with authority.They are beyond questioning. If somebody else is saying [the same thing,] they will ask where is the proof?" This was also the case with a private sector entity that had opened a branch in Delhi. Practitioners in this institution had a high reputation and enjoyed collegiality with allopathic providers across the city, but this could not be generalised to the system of medicine in general.

Political will of highly networked individuals and/or individual access to top decision-makers also facilitated integration. In fact, one of the health system actors had participated in high level negotiations with political leaders in the country to get the AYUSH department formed (formerly the Indian Systems of Medicine & Homeopathy department) in 1995 – which in many ways marks a critical step in the attention given to integration in the health system. Within the state of Delhi, furthermore, it was the demand articulated by city councillors and ward leaders that resulted in the construction of dispensaries and AYUSH wards in hospitals, so much so that this was considered a norm.In Meghalaya, an AYUSH doctor described cordial relations with the administration, such that when medicine stock-outs happened, this officer supplied stop-gap funds to acquire medicines.

Many of the participants we spoke to in Delhi were familiar with each other – these personal relationships and interactions, more often than official platforms, were the basis for interaction, cross-referral, collective planning and advocacy, and in rarer cases, collaborative research. Across

For peer review only - http://bmjopen.bmj.com/site/about/guidelines.xhtml

states, we heard of individual practitioners exercising personal initiative to hasten improvements in infrastructure with the Public Works Department, to increase the visibility of their practice in the facility with their superintendents, and so on.

Personal experience across systems also helped built trust. In Kerala, an allopath indicated that his own mother-in-law was under Ayurvedic treatment for chronic illness and that she and others he knew were "getting good relief." He noted that Ayurveda was trustworthy based on this experience. As an Ayurvedic practitioner in Delhi put it, "if one takes a personal interest, there can be a little something." Indeed for this practitioner, success was measured in much humbler "little somethings" given the larger systemic constraints in the way of integration.

Group or system-linked experiences and meanings –distrust and fragmentation

When speaking about providers as a cadre or group or of systems in general, we noted that difference and distrust tended to be highlighted. In Meghalaya, an allopath opined "Please, if you want us to work in a normal way, you know, peacefully, just have these people removed." A similar sentiment was expressed by a senior Unani hospital practitioner in Delhi, "We can interact as a pathy but our basic concepts do not match. We can't help each other in any way. They are independent, we are independent." There was limitedvalue, in the view of this practitioner, in engaging with other systems of medicine. Among older generations of practitioners, relationships were more fraught, and characterised by inter-system tensions. An allopath in Kerala described at length how allopathic doctors had protested vehemently – and successfully – against a government policy posting of Ayurveda doctors getting house surgeon postings in the state. In contrast, other, usually more junior practitioners, had a high demand for inter and intra-system interaction including but also beyond the official framework of workshops, chances to "sit and talk" about benefits, collaborative research ideas, and so on.

For peer review only - http://bmjopen.bmj.com/site/about/guidelines.xhtml

“Little somethings,” but “nothing as such”

In addition, we observed limited formal communication across systems of medicine. As pointed out by a health system actor: “Doctors generally don’t have meetings but just like that when they sit on the table talk so then such conversations happen.”

In Delhi, chances for such interaction were constrained in the system by the fragmentation of jurisdictions and facilities, but also with respect to how providers were posted at facilities. In this state, co-location did take place, but involved an individual TCA practitioner co-located at multiple sites, while multiple allopaths served at a single site (the biomedical norm). Allopaths had more opportunities, in sheer numbers of people, availability of space and time, to communicate with each other. Given the commensurate lack of people, space and time, allopaths had fewer changes to communicate with TCA providers or TCA providers with each other. In Kerala, the limitations on communication were shaped in particular by the fact that facilities tended to be stand-alone. In Meghalaya, an Ayurveda doctor stated, simply, “I am doing my work, that is completely asocial type, separated, segregated.” There was almost no communication between local health practitioners and others – whether AYUSH or allopath simply because of a lack of systemic acknowledgement and legitimacy given to this workforce.This doctor went on: “very few people that listen to our problem. Because, we are still again you know under the general allopathic doctor, no, like our SS, DMHO even the directorate, at the directorate level, the director so when we post our problem you know, hardly like, they table that problem…”

In addition, as a cadre, AYUSH practitioners had also to contend with dissonance between their expectations and design of service delivery. We observed in many dispensaries and hospitals in Delhi that non-allopathic practitioners were assigned rooms on the top floor of the facility. And most commonly the kinds of cases that they were handling included orthopaedic ailments, and other conditions (motor, neurological, gastric) that constrained mobility and created a very real barrier of access to care within a health care facility for patients.

For peer review only - http://bmjopen.bmj.com/site/about/guidelines.xhtml

"Little somethings," but "nothing as such"

There was also a mismatch in the expectation and provision of diagnostic services, and of human resources. Both homeopathic and Ayurvedic practitioners in Kerala noted the recourse to outsourcing diagnostic investigations because of the lack of facilities in their institutions. Further, there was reliance upon contractual recruitment of human resources to address shortages, which affected the stability and reliability of service delivery, in their view. When we asked an administrator of one of Delhi's newest, state-of-the-art Ayurvedic facilities what kind of coordination occurred across departments as part of the hospital's functioning, he shrugged and replied, "Nothing as such!"

Discussion

Most striking in our findings is the emergence of individual experiences and interpretations as enablers or facilitators of convergence, in the form of collegiality, recognition of stature, exercise of individual agency and cross-referral. These individual efforts were premised on trust and dialogue. In contrast, distrust, poor design and fragmentation at the systems level appears to be a barrier to integrative efforts. It is a system where "little somethings" of individuals that catalyse integration are met with "nothing as such" at the systems level.

Some of our findings are not new – the experience of lack of interaction has emerged in Hollenberg's study on an integrated practice, which reported that weekly doctors' meetings included only biomedical doctors, not CAM.[17]This study also reported the "geographical dominance" of biomedical doctors in terms of location of consulting rooms, as was found in our study. A study by Broom and colleagues found tension, mistrust and dichotomy (rational/irrational, physical/metaphysical, traditional/modern), as well as some inconsistencies in practice, and stated values, regarding biomedicine and TCAM, among Indian oncologists.[18]Such challenges were also seen in our study.

For peer review only - http://bmjopen.bmj.com/site/about/guidelines.xhtml

"Little somethings," but "nothing as such"

Our study also revealed some unique findings with respect to the extant literature. Chung et al, attributed low referral from biomedicine to TCAM in Hong Kongto the lack of articulated and enforced procedures of referral in an integrated medical establishment. In the Indian case, it appears that the vagueness of process both allows ad hoc interactions and referrals based on personal rapport and at the same time discourages the kind of predictable, routine interactions that would allow such rapport to be built.Speaking of integration of Sowa-Rigpa in Bhutan since 1967, Wangchuk and colleagues suggest that there are managerial lessons offered by the juxtaposition and collaboration of conceptually distinct systems within a single administrative and policy unit, such as a ministry.[19]In effect, as they point out, services may not be co-located, but their administration necessarily will be. One could argue that India's case is different – whether in facilities or administratively, it is not just two systems, but more like eight (across AYUSH systems), that are to be integrated, introducing internal hierarchies and complexities that are unique and interlinked.In the 1990s and early 2000s, it was argued that integration is about a "battle between two scientific truths," [20]or that the CAM field creates two tendencies: "uninformed skeptics who don't believe in anything, and uncritical enthusiasts who don't care about data."[21] Analysis of service delivery in India over a decade later suggests that there are multiple battles being fought – epistemological, logistical, ethical, and operational across systems, with (re)conciliatory intercession, at times, of individuals.

Conclusion

Battles occur between armies, while acts of diplomacy involve intricate latticework relationships individuals with overlapping needs and interests. Our research across three very different Indian states – Kerala, Meghalaya and Delhi - suggests that strategies that attempt to make the health systems isomorphic or receptive to individual integrative efforts may facilitate integration across systems, creating opportunities for greater collaboration, and trust. The strategies to this end will

For peer review only - http://bmjopen.bmj.com/site/about/guidelines.xhtml

For peer review only

accordingly need to be individually tailored and carefully devised, so that the system is both more receptive to and reflective of, integrative human agency.

For peer review only

For peer review only - http://bmjopen.bmj.com/site/about/guidelines.xhtml

"Little somethings," but "nothing as such"

Acknowledgements

This researchwas supported by a Wellcome Trust Capacity Strengthening Strategic Award to the Public Health Foundation of India and a consortium of UK universities. We are grateful for the field support of Kaveri Mayra, Candida Thangkhiew, Bobylin Nadon, Darisuk Kharlyngdoh, Ivanhoe Marak, as well as Sabitha Chandran, and the guidance of Dr. Sandra Albert.

Contributorship Statement

Kabir Sheikh and John Porter made substantial contributions to the conception or design of the work; while Devaki Nambiar, Venkatesh Narayan, JK Lakshmi, and TN Sathyanarayana made substantial contributions to the acquisition. All authors substantially contributed to the analysis, and interpretation of data for the work. With Devaki Nambiar playing a lead, coordinating role in drafting the work, all authors revised it critically for important intellectual content, giving final approval of the version to be published. Further, all authors agree to be accountable for all aspects of the work in ensuring that questions related to the accuracy or integrity of any part of the work are appropriately investigated and resolved.

Data Sharing Statement

Unpublished data, in summary form is available to all those who wish to see it. They need only email the lead author.

References

For peer review only

---

[1] World Health Organization. WHO congress on traditional medicine 2008: Beijing declaration. WHO,2011.

2 *Resolution of the 62nd World Health Assembly on Traditional Medicine 2009*. EB124.R9. Geneva: WHA; 2009.

3 Bodeker G, Kronenberg F. A public health agenda for traditional, complementary, and alternative medicine. *Am J Public Health* 2002;92:1582–91.

4 Sheikh K, Nambiar D. Government policies for traditional, complementary and alternative medical services in India: from assimilation to integration? *Natl Med J India* 2011;24:245–6.

[5] McLeod H, Chung VCH. The Integration of Traditional and Complementary Medicine in Health Systems. *Presented at the Joint IACA, IAAHS, and PBSS Colloquium*. Hongkong. 2012. http://www.actuaries.org/HongKong2012/Presentations/TBR11_McLeod.pdf (accessed 9 Sep 2013).

[6] Quaiser N. Essentialising the body. Locating medical domination and resistance. *The Newsletter*. Autumn 2013. http://www.iias.nl/the-newsletter/article/essentialising-body-locating-medical-domination-and-resistance (accessed 21 Jan 2013).

[7] Lohokare M, Davar BV. The Community Role of Indigenous Healers. In: Sheikh K, George A (eds.) *Health Providers in India: On the Frontlines of Change*. New Delhi: Routledge; 2011. p161–81.

[8] Department of Health and Family Welfare. *Mainstreaming AYUSH under NRHM*. http://203.193.146.66/hfw/PDF/ayus.pdf (accessed 31 Mar 2011).

For peer review only - http://bmjopen.bmj.com/site/about/guidelines.xhtml

[9] Department of AYUSH. *AYUSH under NRHM.* http://indianmedicine.nic.in/writereaddata/linkimages/4468616884-AYUSH%20under%20NRHM.pdf (accessed 5 Sep 2013).

[10] Unnikrishnan PM, Kumar LHP, Shankar D. Traditional Orthopaedic Practitioners' Place in Contemporary India: A Case Study from Southern India. In: Sheikh K, George A (eds.) *Health Providers in India: On the Frontlines of Change*. New Delhi: Routledge; 2011. p182–99.

[11] Lakshmi JK. Less equal than others? Experiences of AYUSH medical officers in primary health centres in Andhra Pradesh. *Indian J Med Ethics* 2012;9:18–21.

[12] Priya R, Shweta AS. *Status and Role of AYUSH and Local Health Traditions: Under the National Rural Health Mission, Report of a Study*. National Health Systems Resource Centre. 2010.

[13] Barrett S, Fudge C. *Policy and action: essays on the implementation of public policy*. London: Methuen; 1981.

[14] Walt G, Gilson L. Reforming the health sector in developing countries: the central role of policy analysis. *Health Policy Plan* 1994;9:353–70.

[15]Yanow D. *Conducting Interpretive Policy Analysis*. Newbury Park,CA: Sage; 2000.

[16] Ritchie J, Spencer L. Qualitative Data Analysis for Applied Policy Research. In *Analyzing qualitative data*. London: Routledge;1994. p173-94.

[17] Hollenberg D. Uncharted ground: Patterns of professional interaction among complementary/alternative and biomedical practitioners in integrative health care settings. *Soc Sci Med* 2006;62:731-44.

For peer review only - http://bmjopen.bmj.com/site/about/guidelines.xhtml

---

[18] Broom A, Doron A, Tovey P. The inequalities of medical pluralism: Hierarchies of health, the politics of tradition and the economies of care in Indian oncology. *Soc Sci Med* 2009;69:698–706.

[19] Wangchuk P, Wangchuk D, Aagaard-Hansen J. Traditional Bhutanese medicine (gSo-BA Rig-PA): an integrated part of the formal health care services. *Southeast Asian J Trop Med Public Health* 2007;38:161–7.

[20] Bloom H. *The Lucifer Principle. A Scientific Expedition into the Forces of History*. New York: The Atlantic Monthly Press;1995.

[21] Northridge ME, Mack Jr R. Integrating ethnomedicine into public health. *Am J Public Health* 2002;92:1561-61.

For peer review only - http://bmjopen.bmj.com/site/about/guidelines.xhtml

For peer review only

BMJ Open

Experiences and meanings of integration of TCAM (Traditional, Complementary and Alternative Medical) providers in three Indian states: Results from a cross-sectional, qualitative implementation research study

Journal:	*BMJ Open*
Manuscript ID:	bmjopen-2014-005203.R1
Article Type:	Research
Date Submitted by the Author:	24-May-2014
Complete List of Authors:	Nambiar, Devaki; Public Health Foundation of India,<br>Narayan, Venkatesh; All India Institute of Medical Sciences,<br>Lakshmi, JK; Indian Institute of Public Health - Hyderabad,<br>Porter, John; London School of Hygiene & Tropical Medicine, Department of Clinical Research<br>Sathyanarayana, TN; Indian Institute of Public Health-Bangalore Campus, Health Policy and Management<br>Sheikh, Kabir; Public Health Foundation of India,
**Primary Subject Heading**:	Health services research
Secondary Subject Heading:	Global health, Qualitative research
Keywords:	COMPLEMENTARY MEDICINE, International health services < HEALTH SERVICES ADMINISTRATION & MANAGEMENT, QUALITATIVE RESEARCH

SCHOLARONE™
Manuscripts

For peer review only

Running Head: Experiences and meanings of integration

Title: Experiences and meanings of integration of TCAM (Traditional, Complementary and Alternative Medical) providers in three Indian states: Results from a cross-sectional, qualitative implementation research study

Authors: D Nambiar,[1] VV Narayan,[2] JK Lakshmi,[3] J Porter,[4] TN Sathyanarayana,[33] K Sheikh[q]

1 Public Health Foundation of India, New Delhi, India

2 All India Institute of Medical Sciences, New Delhi, India

3 Indian Institute of Public Health, Hyderabad, India

4 London School of Hygiene and Tropical Medicine, London, United Kingdom

Corresponding Author Information:

Devaki Nambiar, PhD

Research Scientist

Public Health Foundation of India

ISID, 4 Institutional Area

Vasant Kunj 110067

Keywords: Alternative Medicine, Qualitative Evaluation, World Health

Word Count: 3,868868

For peer review only

Abstract:

Objectives: Efforts to engage Traditional Complementary and Alternative Medical (TCAM) practitioners in the public health workforce have growing relevance for India's path to universal health coverage. We used an action-centred framework to understand how policy prescriptions related to integration were being implemented in three distinct Indian states.

Setting: Health departments and district-level primary care facilities in the states of Kerala, Meghalaya, and Delhi.

Participants: In each state, two or three districts were chosen that represented variation in accessibility and distribution across TCAM providers (e.g.,small or large proportions of local health practitioners, Homoeopaths, Ayurvedic and/or Unani practitioners). Per district, two blocks or geographical units were selected. TCAM and allopathic practitioners, administrators and representatives of community at district and state levels were chosen based on publicly available records from state and municipal authorities. A total of 196 interviews were carried out: 74 in Kerala, and 61 each in Delhi and Meghalaya.

Primary and secondary outcome measures: We sought to understand experiences and meanings associated with integration across stakeholders, as well as barriers and facilitators to implementing policies related to integration of TCA providers at the systems level.

Results: We found that individual and interpersonal attributes tended to facilitate integration, while system features and processes tended to hinder it. Collegiality, recognition of stature, exercise of individual personal initiative among TCA practitioners and of personal experience of TCAM among allopaths enabled integration. The system was characterised, on the other hand, by fragmentation of jurisdiction and facilities, inter-system isolation, lack of trust in and awareness of TCA systems, and inadequate infrastructure and resources for TCA service delivery.

For peer review only - http://bmjopen.bmj.com/site/about/guidelines.xhtml

Conclusions: State-tailored strategies that routinise interaction, reward individual and system-level individual integrative efforts, fostered by high level political will are recommended.

Strengths

* Multi-sited qualitative study drawing on meanings and experiences across patients, providers, and health systems administrators

* Implementation research using rigourously applied interpretive policy analysis methods

* Linked to India's path on Universal Health Coverage

Limitations

* Cross-sectional study, so other than self-report of historical changes, we were not able to chart or map changed views or experiences of participants in vivo.

* Focus on the public service delivery sector, even as a great deal of health-seeking takes place in the private sector, with the assumption that public sector strengthening is highly desirable, and possible only through focused study on it.

For peer review only - http://bmjopen.bmj.com/site/about/guidelines.xhtml

Introduction

The 1978 Alma Ata declaration called for traditional medicine, treatments and practices to be "preserved, promoted and communicated widely and appropriately based on the circumstances in each country." Thirty years later, the 2008 Beijing Declaration on Traditional Medicine called for integration of providers into national health systems, recommending systems of qualification, accreditation, regulation and communication (with allopathic providers).[1] These features of the Beijing Declaration were echoed at the 62nd World Health Assembly in 2009, putting out a call to action to United Nations member states to move forward with their plans for integration.[2] The global positioning of Traditional, Complementary, and Alternative Medicine (TCAM) has issued from and tends to imply a central focus on clinical and experimental medicine,[3] yet, recent calls for health systems integration, drawing attention to features like education, accreditation, regulation, and health services provision, draw attentionto the TCAM health workforce.

In earlier work, we have identified three broad trends of integration as it relates to TCA providers: self-regulation with governmental linkage, government regulation and provisioning, and hybrid/parallel models.[4]This links roughly to the WHO nosology, where three models are identified: "tolerant" systems where the national health care system is based entirely on biomedicine but some TCAM practices are legally permissible, "inclusive" systems where TCAM is recognised but not fully integrated into all aspects of healthcare, and "integrative," where TCAM is officially recognised in national drug policy, providers and products are registered and regulated, therapies are widely available and covered under insurance schemes, research and education are widely accessible.[5]

The situation on the ground in India, hybrid in our view, seems in parts to reflect tendencies across WHO categories. The dominance of biomedicine appears to be a critical feature of India's postcolonial health system, even as pre-independence, the TCAM practitioner community had

For peer review only - http://bmjopen.bmj.com/site/about/guidelines.xhtml

played a major role in resisting colonial domination in the practice of (bio)medicine.[6] In part as a response to the reliance on allopathy throughout modern Indian history, there have been strong arguments in favour of the critical role that non-mainstream practitioners play in offering accessible, affordable, and socially acceptable health services to populations.**Error! Bookmark not defined.**[,7,8] A study in Maharashtra reported that the situation of traditional healing as a community function through shared explanatory frameworks across provider and patient are explicitly unlike typical doctor-patient relationships.[9]

In India, one can also find a larger integrative framework, one that mandates "mainstreaming" of codified TCAM in India, collectively referred to as AYUSH, an acronym for Ayurveda, Yoga & Naturopathy, Unani, Siddha, Sowa-Rigpa, and Homoeopathy. The National Rural Health Mission (NRHM), launched in 2005 to fortify public health in rural India, took a particular interest in integrating AYUSH practitioners through facilitation of specialised AYUSH practice, integration of AYUSH practitioners in national health programmes, incorporation of AYUSH modalities in primary health care, strengthening the governance of AYUSH practice, support forfor AYUSH education, establishment of laboratories and research facilities for AYUSH, and providing infrastructural support.[10] Human resource-focused strategies included contractual appointment of AYUSH doctors in Community and Primary Health Centres, appointment of paramedics, compounders, data assistants, and managers to support AYUSH practice, establishment of specialised therapy centres for AYUSH providers,, inclusion of AYUSH doctors in national disease control programmes; and incorporation of AYUSH drugs into community health workers' primary health care kits. A recent report from the AYUSH department reports that NRHM has established AYUSH facilities in co-location with health facilities in many Indian states (notably, not in Kerala, where the stand-alone AYUSH facility is the chosen norm).[11] As of 2012, more than three quarters of India's district hospitals, over half of its Community Health Centres and over a third of India's Primary Health Centres have

For peer review only - http://bmjopen.bmj.com/site/about/guidelines.xhtml

AYUSH co-location, serving about 1.77 million, 3.3 million, and 100,000 rural Indians, respectively.[11]

And yet, even this integration framework has at most an "inclusive" character. This is reflected in findings like "official neglect" of traditional orthopaedic practitioners who have no registration, uniformity in inter-state regulation, or institutionalized medical training.[12]AYUSH doctors contracted to Medical Officer posts in Primary Health Centres (PHCs) in the southern Indian state of Andhra Pradesh report numerous lacunae in the implementation of the mainstreaming initiatives in the National Rural Health Mission (NRHM):[13] job perquisites are not indicated, no benefits or allowances provided for health, housing or education, and compensation packages are much lower than those of allopathic doctors. Support for AYUSH practice is also inadequate (lack of infrastructure, trained assistants, and drug supply) and unethical practices have also been reported (documenting attendance of absentees, and non-cooperation from non-AYUSH personnel).Evidence from NRHM suggests that reshuffled AYUSH providers practice forms of medicine beyond the scope of their training.[14]Paradoxically, moreover, some Indian states prohibit cross-system prescription, adding ethical dilemmas for TCA practitioners who serve as the only medical practitioner in resource-poor areas.[14]

At a larger scale, current practices of integration (as in NRHM) have been described as substitution and replacement; which tend to ignore the merits of TCAM and present more barriers than facilitators of integration.[7] Particularly given the strong push towards co-location and other strategies of integration as part of India's move towards Universal Health Coverage, the integration of AYUSH practitioners could result in a doubling of the health workforce. And yet, there are strong fears that such an emphasis on quantitative aspects of integration, i.e. having the right number of practitioners placed at facilities is inadequate. There is a need to critically and qualitatively appraise the government infrastructure to support TCA, identify barriers and facilitators to integration that have emerged from this rapid placement of these practitioners, and

For peer review only - http://bmjopen.bmj.com/site/about/guidelines.xhtml

how these TCA practitioners, allopathic practitioners, and health system actors are reacting and adapting to each.

Methods

This analysis draws from a larger mixed methods implementation research study aimed at understanding operational and ethical challenges in integration of TCA providers for delivery of essential health services in three Indian states. The study looked at the contents and implementation of TCA provider integration policies in 3 states and at national level examining the understanding and interpretations of integration from the perspectives of different health systems actors. These coupled with their experiences in the actual processes of integration of TCA providers were studied using qualitative interview methods to help identify systemic and ethical challenges. Based on this, the study sought to derive strategies to augment the integration of TCA providers in the delivery of essential health services.

Our study was based on action-centred frameworks[15] with a focus on policy *actors* and *processes*.[16] We have therefore sought to understand the implementation of integration policies empirically. A team of four field researchers was oriented by the principal investigator and advisor to the post-positivist paradigm of research, using Yanow's model of interpretative policy analysis, where the emphasis is equally on describing the experience of policy processes, and on elaborating the meanings actors attach to those processes.[17] The research protocol was approved by the Institutional Ethics Committee of the Public Health Foundation of India.

Our methods included semi-structured in-depth interviews (see interview guides, Appendix 1) with policymakers (N=12), administrators (N=43), TCAM practitioners, (N=59) allopathic practitioners (N=37), traditional healers (N=7), as well as health workers and community representatives (N=38) in three diverse Indian states (see map, Figure 1). We undertook the study in Kerala, where a number of systems have strong historical and systemic roots (N=74),

For peer review only - http://bmjopen.bmj.com/site/about/guidelines.xhtml

Meghalaya, where local health traditions hold sway (N=61), and Delhi, where national, state, and municipal jurisdictions interface with multiple systems of medicine (N=61). Participants were selected based upon maximum variation criteria for each category. We sought to represent different schemes, levels of implementation (directorates, zonal officers), systems of medicine, types of establishments (hospital, dispensary), and years of experience.

In each state, one senior researcher, a research associate and a field researcher developed selection matrices to achieve maximum variation across each category of respondents. In each state, two districts were chosen (in the case of Delhi, three municipal zones) that represented variation in accessibility and distribution across TCA providers (eg. small or large proportions of local health practitioners, HomoeopathsHomoeopaths, Ayurvedic and/or Unani practitioners). Publicly available records from state and municipal authorities were consulted in order to determine location and type of facility (co-located, stand-alone) as well as suggestions and recommendations from Key Informants. We also ensured that facilities closest to and furthest from district headquarters were chosen for interviews, to maximise variability. We would typically contact providers via cell phone, share information about the study verbally or via email, and set up a time to interview them. In some cases, we would arrive during out-patient clinic hours to the chosen facility, share our participant information sheet and seek an appointment time with eligible participants. In most cases, we found that participants were keen to participate once they were aware of the nature of the study and, in some cases, the assurance of confidentiality. We had no refusals, although some allopathic practitioners had to be persuaded to participate by emphasizing that this study was not "pro-TCAM integration" per se, but merely seeking to understand state policy implementation. Interviews, ranging from 15 to 90 minutes in length were undertaken, always with prior informed consent, and separate consent to record interviews. Data transcribed and stored in password-protected folders and each transcript was checked by investigators for accuracy and quality of transcription.

For peer review only - http://bmjopen.bmj.com/site/about/guidelines.xhtml

Textual data from transcripts of interviews as well as notes and observations of facilities and service delivery recorded during fieldwork were analysed through a combination of deductive and inductive techniques in the "framework" approach of qualitative analysis for applied policy research[18]using ATLAS.ti7 software. Themes were developed in three iterations: in the first stage, the lead researcher from each state applied *a priori* codes and closely perused transcripts to devise *emergent* codes, with the support of the Research Associate.RA *A priori* codes were based on our research questions, reflecting experiences, interpretations and meanings of integration (eg. Tc_Ap_El_Adm refers to a TCAM providers' explanation of experience of interactions with administration in the facility or the health care system). *Emergent* codes were used to describe the content or categories of these experiences, interpretations and meanings (eg. Em_El_IndInit refers to personal initiative as a determinant of integration). Researchers coded20% of each other's state datasets to ensure that codes were being applied in a similar, uniform manner. In the second stage, agreement and consolidation of emergent codes across three sites took place under the direction of the study lead; these were then applied to data from each state by its respective lead researcher. Concurrently, lead researchers developed super-codes, or *analytic* codes to group emergent codes (eg. An_Ope_Adhoc refers to adhocism in policies and practices related to integration). The study lead finalised and then indexed across sites to arrive at results. Emergent and analytic code families were used to develop analyses, involving sharing of data and consultation across sites. In this paper, we focus on emergent codes related to experiences and interpretations of integration.

Results

We found that facilitators of integration emerged from individual and interpersonal relationships, while barriers were identified at the systems level (see Table 1).

Facilitators at the individual/interpersonal level

For peer review only - http://bmjopen.bmj.com/site/about/guidelines.xhtml

*A) **Collegiality between practitioners within facilities***

Interpersonal collegiality waswas between and across TCA and allopathic practitioners. In Meghalaya, an allopathic medical officer noted that in some places Ayurvedic and HomoeopathicHo doctors were collaborating closely with his colleagues, expressing an interest in learning more about allopathic practices. In the same state, an AYUSH doctor described cordial relations with the administration, such that when medicine stock-outs happened, the allopathic medical officer supplied stop-gap funds to acquire medicines.

*B) **Stature of TCA doctors***

Another aspect was the "stature" of individual practitioners. In Kerala, an Ayurvedic practitioner noted that: "Nobody can question <Name of Well Known Ayurvedic Physician from Kerala>. IfI he says that taking *chavanaprasham* [health paste] will lead to DNA repair, then nobody can question because they are saying with authority.They are beyond questioning. If somebody else is saying [the same thing,] they will ask, where is the proof?" This was also the case with a private sector entity that had opened a branch in Delhi. Practitioners in this institution were highly reputed, involved with transnational research collaborations, and reported numerous cross-referrals from allopathic providers across the city. .

*C) **Personal initiative of TCA doctors***

Across states, we heard of individual TCA practitioners exercising personal initiative to hasten improvements in infrastructure andand service delivery. Following is an excerpt of an interview with an Ayurvedic doctor from a Delhi hospital: "ThereThere is a lack of storage space so the diagnosis room is being used for some storage. But I have been treating people in the Public Works Department and then it is getting resolved!"!" Many of the participants we spoke to in many states were familiar with each other – these personal relationships and interactions, in the

For peer review only - http://bmjopen.bmj.com/site/about/guidelines.xhtml

absence of official or regular platforms, were the basis for interaction, cross-referral, collective planning and advocacy, and in rarer cases, collaborative research.

D) *Personal experience of allopaths*

Personal experience across systems also helped built trust. In Kerala, an allopath indicated that his own mother-in-law was under Ayurvedic treatment for chronic illness and that she and others he knew were "getting good relief." He noted that Ayurveda was trustworthy based on this experience. As an Ayurvedic practitioner in Delhi put it, "if one takes a personal interest, there can be a little something. But everyone is busy in their own work. If it is done officially – like in a month, every 2nd Saturday …Then it will happen more systematically."

E) *Political will of senior health system actors*

Systems level integration was facilitated by highly networked individuals and/or individual access to top decision-makers. One of the health system actors we interviewed had participated in high level negotiations with political leaders in the country to get the AYUSH department formed (formerly the Indian Systems of Medicine & Homoeopathy department) in 1995 – which in many ways marks a critical step in the attention given to integration in the health system. Within the state of Delhi, furthermore, it was the demand articulated by city councillors and ward leaders that resulted in the construction of dispensaries and AYUSH wards in hospitals, so much so that this was considered a norm.

Barriers at the system level

A) *Fragmentation of jurisdictions and facilities*

It was clear that systematic integration was not widely perceived in any of the facilities or states studied. For one, all states had not a single unified system, but rather multiple systems with parallel governance apparatuses, each with their own challenges. In fact, in Delhi, integration was

For peer review only - http://bmjopen.bmj.com/site/about/guidelines.xhtml

constrained in the system by the fragmentation of jurisdictions and facilities, but also with respect to how providers were posted at facilities. In this state, co-location did take place, but involved an individual TCA practitioner co-located at multiple sites, while multiple allopaths served at a single site (the biomedical norm). Allopaths had more opportunities, in terms of sheer numbers of people, availability of space and time, to communicate with each other.

*B) **Inter-system isolation and lack of communication***

Given the aforementioned lack of people, space and time, allopaths were socially isolated from, and had fewer chances to communicate with TCA providers or TCA providers with each other. In Kerala, the limitations on communication were shaped in particular by the fact that facilities tended to be stand-alone. In Meghalaya, an Ayurveda doctor stated, simply, "I am doing my work, that is completely asocial type, separated, segregated." There was almost no communication between local health practitioners and others – whether AYUSH or allopath simply because of a lack of systemic acknowledgement and legitimacy given to this workforce. This doctor went on: "very few people listen to our problem. Because, we are still, again, you know, under the general allopathic doctor, …so when we post our problem you know, hardly like, they table that problem…"

*C) **Lack of trust and awareness of TCA systems***

When speaking about providers as a cadre, group or systems in general, we noted that distrust tended to be highlighted. In Meghalaya, an allopath opined "Please, if you want us to work in a normal way, you know, peacefully, just have these people removed." A similar sentiment was expressed by a senior Unani hospital practitioner in Delhi, "We can interact as a *pathy* but our basic concepts do not match. We can't help each other in any way. They are independent, we are independent." There was limited value, in the view of this practitioner, in engaging with other systems of medicine. An allopath in Kerala described at length how allopathic doctors had protested vehemently – and successfully – against a government policy of Ayurveda doctors

For peer review only - http://bmjopen.bmj.com/site/about/guidelines.xhtml

getting house surgeon postings in the state. MoreMore junior practitioners noted that even with respect to TCAM systems: "We three [Ayurveda, Unani, and Homoeopathy] are together here, but cross-reference is very, very less…We don't know what is the strong point of Ayurveda, Unani. Allopath will not know the strong point of Homoeopathy, Ayurveda. They just say 'skin!' – that's all they know!"!"

D) ***Inadequate infrastructure and resources for TCA service delivery***

Opportunities to interact were further constrained by the system design of service delivery. We observed in many dispensaries and hospitals in Delhi that non-allopathic practitioners were assigned rooms on the top floor of the facility, while allopaths were allocated multiple rooms on the ground floor (Fieldnotes June 11th, 20th, 21st, 22nd, and 27th 2012). And, most commonly, the kinds of cases that they were handling included orthopaedic ailments, and other conditions (motor, neurological, gastric) that constrained mobility and created a very real barrier of access to care within a health care facility for patients. Practitioners therefore spend much of their time responding to these inadequacies.

Therewere also shortcomings in the design of diagnostic services, and inadequacy of human resources. Both HomoeopathicHo and Ayurvedic practitioners in Kerala noted the recourse to outsourcing diagnostic investigations because of the lack of facilities in their institutions. Further, there was reliance upon contractual recruitment of human resources to address shortages, which affected the stability and reliability of service delivery, in their view. When we asked an administrator of one of Delhi's newest, state-of-the-art Ayurvedic facilities what kind of coordination occurred across departments as part of the hospital's functioning, he shrugged and replied, "Nothing as such!"

For peer review only - http://bmjopen.bmj.com/site/about/guidelines.xhtml

Discussion

Most striking in our findings is the emergence of individual experiences and interpretations as enablers or facilitators of integrationintegration, in the form of collegiality, recognition of stature, exercise of personal initiative among TCA practitioners and of personal experience of TCAM among allopaths. In contrast, barriers to integration seemed to exist at a systems level. They included fragmentation of jurisdiction and facilities, inter-system isolation, lack of trust in and awareness of TCA systems, and inadequate infrastructure and resources for TCA service delivery.delivery It is a system where "little somethings" of individuals that catalyse integration are met with "nothing as such" at the systems level.

Some of our findings are not new – the experience of lack of interaction has emerged in Hollenberg's study on an integrated practice, which reported that weekly doctors' meetings included only biomedical doctors, not CAM.[19]This study also reported the "geographical dominance" of biomedical doctors in terms of location of consulting rooms, as was found in our study. A study by Broom and colleagues found tension and mistrust, as well as inconsistencies in practice and values related to biomedicine and TCAM, among Indian oncologists.[20]Such challenges were also seen in our study.

Our study also revealed some unique findings with respect to the extant literature. Chung et al, attributed low referral from biomedicine to TCAM in Hong Kong to the lack of articulated and enforced procedures of referral in an integrated medical establishment.[21] In the Indian case, it appears that the vagueness of process both allows ad hoc interactions and referrals based on personal rapport and at the same time discourages the kind of predictable, routine interactions that would allow such rapport to be built. Speaking of integration of Sowa-Rigpa in Bhutan since 1967, Wangchuk and colleagues suggest that there are managerial lessons offered by the juxtaposition and collaboration of conceptually distinct systems within a single administrative and policy unit, such as a ministry.[22]In effect, as they point out, services may not be co-located,

For peer review only - http://bmjopen.bmj.com/site/about/guidelines.xhtml

but their administration necessarily should be. One could argue that India's case is different – whether in facilities or administratively, it is not just two systems, but more like eight (across AYUSH systems), that are to be integrated, introducing internal hierarchies and complexities that are unique to the countrycountry.

In the 1990s and early 2000s, it was argued that integration is about a "battle between two scientific truths," [23]or that the CAM field creates two tendencies: "uninformed skeptics who don't believe in anything, and uncritical enthusiasts who don't care about data."[24] Analysis of service delivery in India over a decade later suggests that there are multiple battles being fought – epistemological, logistical, ethical, and operational across systems, with (re)conciliatory intercession, at times, of individuals.

How can such intercessions be encouraged, catalyzed even? We offer a few suggestions for activities in the Indian case that leverage the individual facilitators of integration to fill systemic gaps (see Table 2). These strategies are based on the aforementioned findings in particular states; their 'translate-ability' to other states would have to be examined.

For one, improved documentation of clinical cases across systems could be undertaken and shared. We noted that those AYUSH practitioners who were documenting their practices had greater stature, opportunities and topics for interaction with peers. Drawing upon personal initiative and creating experiences of interaction, this could help raise the stature of TCA practice, while also reducing isolation and lack of awareness. State health departments could create routine opportunities for interaction and collaboration across systems, and within facilities. In Delhi, polio immunization has served as an integrative platform for many practitioners to work together and develop trust and ties. Within facilities, joint staff meetings may serve a similar purpose. Authorities may also consider rewarding individual initiatives for integration (through challenge grants or institutional recognition) - these could be designed to address system-level barriers to integration. Systems-integration could also be rewarded, through

For peer review only - http://bmjopen.bmj.com/site/about/guidelines.xhtml

joint or synergistically achieved targets for referrals, or number of patients cared for using complementary or adjuvant therapies. As of now, those reporting cross-referrals only know of each other; if targets were set, there would be greater incentives for and attention to conditions and protocols for cross-referral. Many practitioners we spoke to suggested that guidelines for collaboration (including cross-referral) be created. We feel this itself could be a starting point of collaboration amongst TCA providers and with allopathic providers. In each state, the feasibility of each of these strategies would have to be determined, and given due attention through the exertions of powerful stakeholders with political will, who at various points, may find themselves battling each other over policies or power.

Conclusion

Battles occur between armies, while acts of diplomacy involve intricate latticework relationships individuals with overlapping needs and interests. Our research across three very different Indian states – Kerala, Meghalaya and Delhi - suggests that strategies that attempt to make the health systems receptive to individual integrative efforts may facilitate integration across systems, creating opportunities for greater collaboration, and trust. We have proposed strategies to this end, which must in turn be additionally tailored toto each state context, so that the health system exists in a vibrant but also coherent plurality of human agency.

For peer review only - http://bmjopen.bmj.com/site/about/guidelines.xhtml

Tables

Table 1. Summary of Findings

	Factors at Individual/Interpersonal Level		Factors at Group/System Level
**FACILITATORS**	A) **Collegiality** between practitioners within facilities<br>B) **Stature** of TCA doctors<br>C) **Personal initiative** of TCA doctors<br>D) **Personal experience** of allopaths<br>E) **Political will** of senior health system actors	**BARRIERS**	A) **Fragmentation** of jurisdiction and facilities<br>B) Inter-system **isolation** and lack of communication<br>C) Lack of **trust and awareness** of TCA systems<br>D) **Inadequate infrastructure and resources** for TCA service delivery

For peer review only - http://bmjopen.bmj.com/site/about/guidelines.xhtml

Table 2. Recommendations to promote/address Integration, responding to findings

	FACILITATORS				BARRIERS			
**Strategies to promote TCA integration for essential health services delivery, based on our findings**	Collegiality	Stature	Personal Initiative	Personal Experience	Fragmentation	Isolation	Lack of trust/ awareness	Inadequate infrastructure/ resources
**High level political will required for all strategies**								
Case documentation and sharing across systems, and in the academic literature		+	+	+		+	+	
Routine opportunities for interaction and collaboration across systems (eg. health camps, health promotion drives)	+			+	+	+	+	+
Routine opportunities for interaction within co-located facilities (eg. staff meetings)	+			+	+	+	+	+
Rewards for integrative initiative of individuals (eg. challenge grants or institutional recognition)	+	+	+	+				
Rewards for integrative initiative at systems or facility level (eg. Joint targets like no of monthly referrals, no of cases jointly resolved)		+		+	+	+	+	+
Guidelines for collaboration (criteria and conditions for cross-referral, jointly developed by practitioners, non-clinical aspects of work together, including health promotion and managerial duties)				+	+	+	+	+

For peer review only

For peer review only - http://bmjopen.bmj.com/site/about/guidelines.xhtml

Acknowledgements

This research was supported by a Wellcome Trust Capacity Strengthening Strategic Award to the Public Health Foundation of India and a consortium of UK universities. We are grateful for the field support of Kaveri Mayra, Candida Thangkhiew, Bobylin Nadon, Darisuk Kharlyngdoh, Ivanhoe Marak, as well as Sabitha Chandran, and the guidance of Dr. Sandra Albert.

For peer review only

Contributorship statement

Kabir Sheikh and John Porter made substantial contributions to the conception or design of the work; while Devaki Nambiar, Venkatesh Narayan, JK Lakshmi, and TN Sathyanarayana made substantial contributions to the acquisition. All authors substantially contributed to the analysis, and interpretation of data for the work. With Devaki Nambiar playing a lead, coordinating role in drafting the work, all authors revised it critically for important intellectual content, giving final approval of the version to be published. Further, all authors agree to be accountable for all aspects of the work in ensuring that questions related to the accuracy or integrity of any part of the work are appropriately investigated and resolved.

Funding

This research was supported by a Wellcome Trust Capacity Strengthening Strategic Award to the Public Health Foundation of India and a consortium of UK universities.

Competing interests

None to declare.

Data sharing

No additional data available.

For peer review only - http://bmjopen.bmj.com/site/about/guidelines.xhtml

References

[1] World Health Organization. WHO congress on traditional medicine 2008: Beijing declaration. WHO,2011.

2 *Resolution of the 62nd World Health Assembly on Traditional Medicine 2009*. EB124.R9. Geneva: WHA; 2009.

3 Bodeker G, Kronenberg F. A public health agenda for traditional, complementary, and alternative medicine. *Am J Public Health* 2002;92:1582–91.

4 Sheikh K, Nambiar D. Government policies for traditional, complementary and alternative medical services in India: from assimilation to integration? *Natl Med J India* 2011;24:245–6.

[5] McLeod H, Chung VCH. The Integration of Traditional and Complementary Medicine in Health Systems. *Presented at the Joint IACA, IAAHS, and PBSS Colloquium*. Hongkong. 2012. http://www.actuaries.org/HongKong2012/Presentations/TBR11_McLeod.pdf (accessed 9 Sep 2013).

[6] Quaiser N. Essentialising the body. Locating medical domination and resistance. *The Newsletter*. Autumn 2013. http://www.iias.nl/the-newsletter/article/essentialising-body-locating-medical-domination-and-resistance (accessed 21 Jan 2013).

[7] Khan S. Systems of medicine and nationalist discourse in India: Towards "new horizons" in medical anthropology and history. *SocSci Med*. 2006;62: 2786-97.

[8] World Health Organization. WHO Traditional Medicine Strategy 2002 – 2005. WHO,2002.

[9] Lohokare M, Davar BV. The Community Role of Indigenous Healers. In: Sheikh K, George A (eds.) *Health Providers in India: On the Frontlines of Change*. New Delhi: Routledge; 2011. p161–81.

For peer review only - http://bmjopen.bmj.com/site/about/guidelines.xhtml

[10] Department of Health and Family Welfare. *Mainstreaming AYUSH under NRHM.* http://203.193.146.66/hfw/PDF/ayus.pdf (accessed 31 Mar 2011).

[11] Department of AYUSH. *AYUSH under NRHM.* http://indianmedicine.nic.in/writereaddata/linkimages/4468616884-AYUSH%20under%20NRHM.pdf (accessed 5 Sep 2013).

[12] Unnikrishnan PM, Kumar LHP, Shankar D. Traditional Orthopaedic Practitioners' Place in Contemporary India: A Case Study from Southern India. In: Sheikh K, George A (eds.) *Health Providers in India: On the Frontlines of Change*. New Delhi: Routledge; 2011. p182–99.

[13] Lakshmi JK. Less equal than others? Experiences of AYUSH medical officers in primary health centres in Andhra Pradesh. *Indian J Med Ethics* 2012;9:18–21.

[14] Priya R, Shweta AS. *Status and Role of AYUSH and Local Health Traditions: Under the National Rural Health Mission, Report of a Study*. National Health Systems Resource Centre. 2010.

[15] Barrett S, Fudge C. *Policy and action: essays on the implementation of public policy*. London: Methuen; 1981.

[16] Walt G, Gilson L. Reforming the health sector in developing countries: the central role of policy analysis. *Health Policy Plan* 1994;9:353–70.

[17]Yanow D. *Conducting Interpretive Policy Analysis*. Newbury Park,CA: Sage; 2000.

[18] Ritchie J, Spencer L. Qualitative Data Analysis for Applied Policy Research. In *Analyzing qualitative data*. London: Routledge;1994. p173-94.

For peer review only - http://bmjopen.bmj.com/site/about/guidelines.xhtml

[19] Hollenberg D. Uncharted ground: Patterns of professional interaction among complementary/alternative and biomedical practitioners in integrative health care settings. *Soc Sci Med* 2006;62:731-44.

[20] Broom A, Doron A, Tovey P. The inequalities of medical pluralism: Hierarchies of health, the politics of tradition and the economies of care in Indian oncology. *Soc Sci Med* 2009;69:698–706.

[21] Chung VC, Hillier S, Lau CH, et. al. Referral to and attitude towards traditional Chinese medicine amongst western medical doctors in postcolonial Hong Kong. *Soc Sci Med* 2011;*72*:247-55.

[22] Wangchuk P, Wangchuk D, Aagaard-Hansen J. Traditional Bhutanese medicine (gSo-BA Rig-PA): an integrated part of the formal health care services. *Southeast Asian J Trop Med Public Health* 2007;38:161–7.

[23] Bloom H. *The Lucifer Principle. A Scientific Expedition into the Forces of History*. New York: The Atlantic Monthly Press;1995.

[24] Northridge ME, Mack Jr R. Integrating ethnomedicine into public health. *Am J Public Health* 2002;92:1561-61.

For peer review only - http://bmjopen.bmj.com/site/about/guidelines.xhtml

Experiences and meanings of integration

Running Head: Experiences and meanings of integration

Title: Experiences and meanings of integration of TCAM (Traditional, Complementary and Alternative Medical) providers in three Indian states: Results from a cross-sectional, qualitative implementation research study

Authors: D Nambiar,[1] VV Narayan,[2] JK Lakshmi,[3] J Porter,[4] TN Sathyanarayana,[335] K Sheikh[q]

1 Public Health Foundation of India, New Delhi, India

2 All India Institute of Medical Sciences, New Delhi, India

3 Indian Institute of Public Health, Hyderabad, India

4 London School of Hygiene and Tropical Medicine, London, United Kingdom

~~5 Indian Institute of Public Health,~~ Hyderabad, ~~Bangalore, India~~

Corresponding Author Information:

Devaki Nambiar, PhD

Research Scientist

Public Health Foundation of India

ISID, 4 Institutional Area

Vasant Kunj 110067

Keywords: Alternative Medicine, Qualitative Evaluation, World Health

Word Count: 3,868868~~075~~

For peer review only - http://bmjopen.bmj.com/site/about/guidelines.xhtml

Acknowledgements

This research was supported by a Wellcome Trust Capacity Strengthening Strategic Award to the Public Health Foundation of India and a consortium of UK universities. We are grateful for the field support of Kaveri Mayra, Candida Thangkhiew, Bobylin Nadon, Darisuk Kharlyngdoh, Ivanhoe Marak, as well as Sabitha Chandran, and the guidance of Dr. Sandra Albert.

For peer review only

Strengths

* Multi-sited qualitative study drawing on meanings and experiences across patients, providers, and health systems administrators

* Implementation research using rigourously applied interpretive policy analysis methods

* Linked to India's path on Universal Health Coverage

Limitations

* Cross-sectional study, so other than self-report of historical changes, we were not able to chart or map changed views or experiences of participants in vivo.

* Focus on the public service delivery sector, even as a great deal of health-seeking takes place in the private sector, with the assumption that public sector strengthening is highly desirable, and possible only through focused study on it.

Competing Interests

None declared.

For peer review only - http://bmjopen.bmj.com/site/about/guidelines.xhtml

Experiences and meanings of integration

Contributorship statement

Kabir Sheikh and John Porter made substantial contributions to the conception or design of the work; while Devaki Nambiar, Venkatesh Narayan, JK Lakshmi, and TN Sathyanarayana made substantial contributions to the acquisition. All authors substantially contributed to the analysis, and interpretation of data for the work. With Devaki Nambiar playing a lead, coordinating role in drafting the work, all authors revised it critically for important intellectual content, giving final approval of the version to be published. Further, all authors agree to be accountable for all aspects of the work in ensuring that questions related to the accuracy or integrity of any part of the work are appropriately investigated and resolved.

Competing interests

None to declare.

Funding

This research was supported by a Wellcome Trust Capacity Strengthening Strategic Award to the Public Health Foundation of India and a consortium of UK universities.

Data sharing

No additional data available.

For peer review only - http://bmjopen.bmj.com/site/about/guidelines.xhtml

~~Experiences and meanings of integration~~

Introduction

The 1978 Alma Ata declaration called for traditional medicine, treatments and practices to be "preserved, promoted and communicated widely and appropriately based on the circumstances in each country." Thirty years later, the 2008 Beijing Declaration on Traditional Medicine called for integration of providers into national health systems, recommending systems of qualification, accreditation, regulation and communication (with allopathic providers).[1] These features of the Beijing Declaration were echoed at the 62nd World Health Assembly in 2009, putting out a call to action to United Nations member states to move forward with their plans for integration.[2] The global positioning of Traditional, Complementary, and Alternative Medicine (TCAM) has issued from and tends to imply a central focus on clinical and experimental medicine,[3] yet, recent calls for health systems integration, drawing attention to features like education, accreditation, regulation, and health services provision, ~~place~~ draw ~~greater~~ attention ~~upon~~ to the TCAM health workforce.

In earlier work, we have identified three broad trends of integration as it relates to TCA providers: self-regulation with governmental linkage, government regulation and provisioning, and hybrid/parallel models.[4]This links roughly to the WHO nosology, where three models are identified: "tolerant" systems where the national health care system is based entirely on biomedicine but some TCAM practices are legally permissible, "inclusive" systems where TCAM is recognised but not fully integrated into all aspects of healthcare, and "integrative," where TCAM is officially recognised in national drug policy, providers and products are registered and regulated, therapies are widely available and covered under insurance schemes, research and education are widely accessible.[5]

The situation on the ground in India, hybrid in our view, seems in parts to reflect tendencies across WHO categories. The dominance of biomedicine appears to be a critical feature of India's

For peer review only - http://bmjopen.bmj.com/site/about/guidelines.xhtml

Experiences and meanings of integration

postcolonial health system, even as pre-independence, the TCAM practitioner community had played a major role in resisting colonial domination in the practice of (bio)medicine.[6] In part as a response to the reliance on allopathy throughout modern Indian history, there have been strong arguments in favour of the critical role that non-mainstream practitioners play in offering accessible, affordable, and socially acceptable health services to populations.**Error! Bookmark not defined.**[4,7,8] A study in Maharashtra reported that the situation of traditional healing as a community function through shared explanatory frameworks across provider and patient are explicitly unlike typical doctor-patient relationships.[9]

In India, one can also find a larger integrative framework, one that mandates "mainstreaming" of codified TCAM in India, collectively referred to as AYUSH, an acronym for Ayurveda, Yoga & Naturopathy, Unani, Siddha, Sowa-Rigpa, and Homoeopathy. The National Rural Health Mission (NRHM), launched in 2005 to fortify public health in rural India, took a particular interest in integrating AYUSH practitioners through facilitation of specialised AYUSH practice, integration of AYUSH practitioners in national health programmes, integration incorporation of AYUSH modalities in primary health care, strengthening the governance of AYUSH practice, support forforing AYUSH education, establishment of ing laboratories and research facilities for AYUSH, and providing infrastructural support.[10] Human resource-focused strategies included contractual appointment of AYUSH doctors in Community and Primary Health Centres, appointment of paramedics, compounders, data assistants, and managers to support AYUSH practice, the establishment of specialised therapy centres for AYUSH providers,; inclusion of AYUSH doctors in national disease control programmes; and incorporation of AYUSH drugs into community health workers' primary health care kits. A recent report from the AYUSH department reports that NRHM has established AYUSH facilities in co-location with health facilities in many Indian states (notably, not in Kerala, where the stand-alone AYUSH facility is the chosen norm).[1111] As of 2012, more than three quarters of India's district hospitals, over half

Formatted: Endr

For peer review only - http://bmjopen.bmj.com/site/about/guidelines.xhtml

of its Community Health Centres and over a third of India's Primary Health Centres have AYUSH co-location, serving about 1.77 million, 3.3 million, and 100,000 rural Indians, respectively.[11]

And yet, even this integration framework has at most an "inclusive" character. This is reflected in findings like "official neglect" of traditional orthopaedic practitioners who have no registration, uniformity in inter-state regulation, or institutionalized medical training.[12]AYUSH doctors contracted to Medical Officer posts in Primary Health Centres (PHCs) in the southern Indian state of Andhra Pradesh report numerous lacunae in the implementation of the mainstreaming initiatives in the National Rural Health Mission (NRHM):[13] job perquisitesprerequisites are not indicated, no benefits or allowances provided for health, housing or education, and compensation packages are much lower than those of allopathic doctors. Support for AYUSH practice is also inadequate (lack of infrastructure, trained assistants, and drug supply) and unethical practices have also been reported (documenting attendance of absentees, and non-cooperation from non-AYUSH personnel refusal to collaborate).Evidence from NRHM suggests that reshuffled AYUSH providers practice forms of medicine beyond the scope of their training.[14]Paradoxically, moreover, some Indian states prohibit cross-system prescription, adding ethical dilemmas for TCA practitioners who serve as the only medical practitioner in resource-poor areas.[1414 26]

At a larger scale, current practices of integration (as in NRHM) have been described as substitution and replacement; which tend to ignore the merits of TCAM and present more barriers than facilitators of integration.[77 40]Particularly given the strong push towards co-location and other strategies of integration as part of India's move towards Universal Health Coverage, however, the integration of AYUSH practitioners could result in a doubling of the health workforce. And yet, there are strong fears that such an emphasis on quantitative aspects of integration, i.e. having the right number of practitioners placed at facilities is inadequate. There is

For peer review only

~~Experiences and meanings of integration~~

a need to critically and qualitatively appraise the government infrastructure to support TCA, identify barriers and facilitators to integration that have emerged from this rapid placement of these practitioners, and how these TCA practitioners, allopathic practitioners, and health system actors are reacting and adapting to each.

Methods

This analysis draws from a larger mixed methods implementation research study aimed at understanding operational and ethical challenges in integration of TCA providers for delivery of essential health services in three Indian states. The study looked at the contents and implementation of TCA provider integration policies in 3 states and at national level examining the understanding and interpretations of integration from the perspectives of different health systems actors. These coupled with their experiences in the actual processes of integration of TCA providers were studied using qualitative interview methods to help identify systemic and ethical challenges. Based on this, the study sought to derive strategies to augment the integration of TCA providers in the delivery of essential health services.

Our study was based on action-centred frameworks[15] with a focus on policy *actors* and *processes*.[16] We have therefore sought to understand the implementation of integration policies empirically. A team of four field researchers was oriented by the principal investigator and advisor to the post-positivist paradigm of research, using Yanow's model of interpretative policy analysis, where the emphasis is equally on describing the experience of policy processes, and on elaborating the meanings actors attach to those processes.[17] The research protocol was approved by the Institutional Ethics ~~Review Board~~ Committee of the Public Health Foundation of India.

Our methods included semi-structured in-depth interviews (see interview guides, Appendix 1) with policymakers (N=12), administrators (N=43), TCAM practitioners, (N=59) ~~and~~ allopathic practitioners (N=37), traditional healers (N=7), as well as health workers and community

For peer review only - http://bmjopen.bmj.com/site/about/guidelines.xhtml

~~Experiences and meanings of integration~~

representatives (N=38) in three diverse Indian states (see map, Figure 1). We undertook the study in ~~:~~ Kerala, where a number of systems have strong historical and systemic roots (N=74), Meghalaya, where local health traditions hold sway (N=61), and Delhi, where national, state, and municipal jurisdictions interface with multiple systems of medicine (N=61). Participants were selected based upon maximum variation criteria for each category. We sought to represent different schemes, levels of implementation (directorates, zonal officers), systems of medicine, types of establishments (hospital, dispensary), and years of experience.

In each state, one senior researcher, a research associate and a field researcher developed selection matrices to achieve maximum variation across each category of respondents. In each state, two districts were chosen (in the case of Delhi, three municipal zones) that represented variation in accessibility and distribution across TCA providers (eg. small or large proportions of local health practitioners, Homoeopaths~~homeopaths~~Homoeopaths, Ayurvedic and/or Unani practitioners). Publicly available records from state and municipal authorities were consulted in order to determine location and type of facility (co-located, stand-alone) as well as suggestions and recommendations from Key Informants. We also ensured that facilities closest to and furthest from district headquarters were chosen for interviews, to maximise variability. We would typically contact providers via cell phone, share information about the study verbally or via email, and set up a time to interview them. In some cases, we would arrive during out-patient clinic hours to the chosen facility, share our participant information sheet and seek an appointment time with eligible participants. In most cases, we found that participants were keen to participate once they were aware of the nature of the study and, in some cases, the assurance of confidentiality. We had no refusals, although some allopathic practitioners had to be persuaded to participate by emphasizing that this study was not "pro-TCAM integration" per se, but merely seeking to understand state policy implementation. Interviews, ranging from 15 to 90 minutes in length ~~-~~were undertaken~~,~~ ~~only~~ always with prior informed consent, and separate consent to

For peer review only - http://bmjopen.bmj.com/site/about/guidelines.xhtml

For peer review only

~~Experiences and meanings of integration~~

record interviews. -Data ~~were were~~ transcribed and stored in password-protected folders and each transcript was checked by investigators for ~~corrections~~ accuracy and quality of transcription.

Textual data from transcripts of interviews as well as notes and observations of facilities and service delivery recorded during fieldwork were analysed through a combination of deductive and inductive techniques in the "framework" approach of qualitative analysis for applied policy research[18]using ATLAS.ti7 software. Themes were developed in three iterations: in the first stage, the lead researcher from each state applied *a priori* codes and closely perused transcripts to devise *emergent* codes, with the support of the Research Associate.R~~research~~ A~~associate.~~ *A priori* codes were based on our research questions, reflecting experiences, interpretations and meanings of integration (eg. Tc_Ap_El_Adm refers to a TCAM providers' explanation of experience of interactions with administration in the facility or the health care system). *Emergent* codes were used to describe the content or categories of these experiences, interpretations and meanings (eg. Em_El_IndInit refers to personal initiative as a determinant of integration). Researchers coded ~~part~~ 20% of each other's state datasets to ensure that codes were being applied in a similar, uniform manner. In the second stage, agreement and consolidation of emergent codes across three sites took place under the direction of the study lead; these were then applied to data from each state by its respective lead researcher. Concurrently, lead researchers developed super-codes, or *analytic* codes to group emergent codes (eg. An_Ope_Adhoc refers to adhocism in policies and practices related to integration). The study lead finalised and then indexed across sites to arrive at results. Emergent and analytic code families were used to develop analyses, involving sharing of data and consultation across sites. ~~We~~ In this paper, we focus on ~~present~~ emergent codes related to experiences and interpretations of integration.

Results

~~*Individual experiences and meanings – collaboration and trust*~~

For peer review only - http://bmjopen.bmj.com/site/about/guidelines.xhtml

~~Experiences and meanings of integration~~

We found that facilitators of integration emerged from individual and interpersonal relationships, while barriers were identified at the systems level (see Table 1).

Facilitators at the individual/interpersonal level

A) Collegiality between practitioners within facilities

Interpersonal collegiality was~~collaboration and trust appeared to be features of individual and interpersonal relationships across providers and system actors. For instance, many~~ was ~~reported collegiality~~ between and across TCA and allopathic practitioners. In Meghalaya, an allopathic medical officer noted that in some places Ayurvedic and HomoeopathicH~~hom~~o~~eopathic~~ doctors were collaborating closely with his colleagues, expressing an interest in learning more about allopathic practices. In the same state, an AYUSH doctor described cordial relations with the administration, such that when medicine stock-outs happened, the allopathic medical officer supplied stop-gap funds to acquire medicines.

B) Stature of TCA doctors

Another aspect was the "stature" of individual practitioners. In Kerala, an Ayurvedic practitioner noted that: "Nobody can question ~~MSV~~ <Name of Well Known Ayurvedic Physician from Kerala>. IfI~~if~~ he says that taking *chavanaprasham* [health paste] will lead to DNA repair, then nobody can question because they are saying with authority.They are beyond questioning. If somebody else is saying [the same thing,] they will ask, where is the proof?" This was also the case with a private sector entity that had opened a branch in Delhi. Practitioners in this institution ~~had a high~~ were highly reputed, involved with transnational research collaborations, and reported numerous cross-~~reputation and enjoyed collegiality~~ referrals from ~~with~~ allopathic providers across the city. ~~., but this could not be generalised to the system of medicine in general.~~

C) Personal initiative of TCA doctors

For peer review only - http://bmjopen.bmj.com/site/about/guidelines.xhtml

Experiences and meanings of integration

Political will of highly networked individuals and/or individual access to top decision-makers also facilitated integration. In fact, one of the health system actors had participated in high level negotiations with political leaders in the country to get the AYUSH department formed (formerly the Indian Systems of Medicine & Homoeopathy department) in 1995 – which in many ways marks a critical step in the attention given to integration in the health system. Within the state of Delhi, furthermore, it was the demand articulated by city councillors and ward leaders that resulted in the construction of dispensaries and AYUSH wards in hospitals, so much so that this was considered a norm. In Meghalaya, an AYUSH doctor described cordial relations with the administration, such that when medicine stock-outs happened, this the allopathic medical officer supplied stop-gap funds to acquire medicines.

Many of the participants we spoke to in Delhi were familiar with each other – these personal relationships and interactions, more often than official platforms, were the basis for interaction, cross-referral, collective planning and advocacy, and in rarer cases, collaborative research. Across states, we heard of individual TCA practitioners exercising personal initiative to hasten improvements in infrastructure andwith the Public Works Departmentand service delivery. Following is an excerpt of an interview with an Ayurvedic doctor from a Delhi hospital: "ThereTthere is a lack of storage space so the diagnosis room is being used for some storage. But I have been treating people in the Public Works Department and then it is getting resolved!"!";. Many of the participants we spoke to in many states were familiar with each other – these personal relationships and interactions, in the absence of official or regular platforms, were the basis for interaction, cross-referral, collective planning and advocacy, and in rarer cases, collaborative research. to increase the visibility of their practice in the facility with their superintendents, and so on.

*D) **Personal experience of allopaths***

Experiences and meanings of integration

Personal experience across systems also helped built trust. In Kerala, an allopath indicated that his own mother-in-law was under Ayurvedic treatment for chronic illness and that she and others he knew were "getting good relief." He noted that Ayurveda was trustworthy based on this experience. As an Ayurvedic practitioner in Delhi put it, "if one takes a personal interest, there can be a little something. ." Indeed for this practitioner, success was measured in much humbler "little somethings" given the larger systemic constraints in the way of integration. But everyone is busy in their own work. If it is done officially – like in a month, every 2nd Saturday …Then it will happen more systematically."

*E) **Political will of senior health system actors***

Systems level integration was facilitated by highly networked individuals and/or individual access to top decision-makers. One of the health system actors we interviewed had participated in high level negotiations with political leaders in the country to get the AYUSH department formed (formerly the Indian Systems of Medicine & Homoeopathy department) in 1995 – which in many ways marks a critical step in the attention given to integration in the health system. Within the state of Delhi, furthermore, it was the demand articulated by city councillors and ward leaders that resulted in the construction of dispensaries and AYUSH wards in hospitals, so much so that this was considered a norm.

Barriers at the system level

*A) **Fragmentation of jurisdictions and facilities***

Group or system-linked experiences and meanings – distrust and fragmentation

It was clear that systematic integration was not widely perceived in any of the facilities or states studied. For one, all states had not a single unified system, but rather multiple systems with

For peer review only

~~Experiences and meanings of integration~~

parallel governance apparatuses, each with their own challenges. In fact, in Delhi, ~~In Delhi,~~ integration was constrained in the system by the fragmentation of jurisdictions and facilities, but also with respect to how providers were posted at facilities. In this state, co-location did take place, but involved an individual TCA practitioner co-located at multiple sites, while multiple allopaths served at a single site (the biomedical norm). Allopaths had more opportunities, in terms of sheer numbers of people, availability of space and time, to communicate with each other.

B) *Inter-system isolation and lack of communication*

Given the aforementioned lack of people, space and time, allopaths were socially isolated from, and had fewer chances to communicate with TCA providers or TCA providers with each other. In Kerala, the limitations on communication were shaped in particular by the fact that facilities tended to be stand-alone. In Meghalaya, an Ayurveda doctor stated, simply, "I am doing my work, that is completely asocial type, separated, segregated." There was almost no communication between local health practitioners and others – whether AYUSH or allopath simply because of a lack of systemic acknowledgement and legitimacy given to this workforce. This doctor went on: "very few people listen to our problem. Because, we are still, again, you know, under the general allopathic doctor, …so when we post our problem you know, hardly like, they table that problem…"

C) *Lack of trust and awareness of TCA systems*

When speaking about providers as a cadre, ~~or~~ group or ~~of~~ systems in general, we noted that ~~difference and~~ distrust tended to be highlighted. In Meghalaya, an allopath opined "Please, if you want us to work in a normal way, you know, peacefully, just have these people removed." A similar sentiment was expressed by a senior Unani hospital practitioner in Delhi, "We can interact as a *pathy* but our basic concepts do not match. We can't help each other in any way. They are independent, we are independent." There was limited value, in the view of this

For peer review only

Experiences and meanings of integration

practitioner, in engaging with other systems of medicine. Among older generations of practitioners, relationships were more fraught, and characterised by inter-system tensions. An allopath in Kerala described at length how allopathic doctors had protested vehemently – and successfully – against a government policy posting of Ayurveda doctors getting house surgeon postings in the state. MoreIn contrast, other, usually moreMore junior practitioners noted that even with respect to TCAM systems: "We three [Ayurveda, Unani, and Homoeopathy] are together here, but cross-reference is very, very less…We don't know what is the strong point of Ayurveda, Unani. Allopath will not know the strong point of Homoeopathy, Ayurveda. They just say 'skin!' – that's all they know!"!", had a high demand for inter and intra-system interaction including but also beyond the official framework of workshops, chances to "sit and talk" about benefits, collaborative research ideas, and so on.

*D) **Inadequate infrastructure and resources for TCA service delivery***

In addition, we observed limited formal communication across systems of medicine. As pointed out by a health system actor: "Doctors generally don't have meetings but just like that when they sit on the table, talk, so then such conversations happen."

In Delhi, chances for such interaction were constrained in the system by the fragmentation of jurisdictions and facilities, but also with respect to how providers were posted at facilities. In this state, co-location did take place, but involved an individual TCA practitioner co-located at multiple sites, while multiple allopaths served at a single site (the biomedical norm). Allopaths had more opportunities, in sheer numbers of people, availability of space and time, to communicate with each other. Given the commensurate lack of people, space and time, allopaths had fewer changes to communicate with TCA providers or TCA providers with each other. In Kerala, the limitations on communication were shaped in particular by the fact that facilities tended to be stand-alone. In Meghalaya, an Ayurveda doctor stated, simply, "I am doing my work, that is completely asocial type, separated, segregated." There was almost no

For peer review only - http://bmjopen.bmj.com/site/about/guidelines.xhtml

Experiences and meanings of integration

communication between local health practitioners and others – whether AYUSH or allopath simply because of a lack of systemic acknowledgement and legitimacy given to this workforce.This doctor went on: "very few people that listen to our problem. Because, we are still again you know under the general allopathic doctor, no, like our SS, DMHO even the directorate, at the directorate level, the director so when we post our problem you know, hardly like, they table that problem…"

Opportunities to interact were further constrained by the In addition, as a cadre, AYUSH practitioners had also to contend with dissonance between their expectations and system design of service delivery. We observed in many dispensaries and hospitals in Delhi that non-allopathic practitioners were assigned rooms on the top floor of the facility, while allopaths were allocated multiple rooms on the ground floor (Fieldnotes June 11th, 20th, 21st, 22nd, and 27th 2012). And, most commonly, the kinds of cases that they were handling included orthopaedic ailments, and other conditions (motor, neurological, gastric) that constrained mobility and created a very real barrier of access to care within a health care facility for patients. Practitioners therefore spend much of their time responding to these inadequacies.

ThereThere was also a mismatch in the expectation and provision were also shortcomings in the design of diagnostic services, and inadequacy of human resources. Both HomoeopathicHhomoeopathic and Ayurvedic practitioners in Kerala noted the recourse to outsourcing diagnostic investigations because of the lack of facilities in their institutions. Further, there was reliance upon contractual recruitment of human resources to address shortages, which affected the stability and reliability of service delivery, in their view. When we asked an administrator of one of Delhi's newest, state-of-the-art Ayurvedic facilities what kind of coordination occurred across departments as part of the hospital's functioning, he shrugged and replied, "Nothing as such!"

For peer review only - http://bmjopen.bmj.com/site/about/guidelines.xhtml

~~Experiences and meanings of integration~~

Discussion

Most striking in our findings is the emergence of individual experiences and interpretations as enablers or facilitators of integration~~convergence~~integration, in the form of collegiality, recognition of stature, exercise of ~~individual~~ personal initiative among TCA practitioners and of personal experience of TCAM among allopaths. ~~agency and cross-referral. These individual efforts were premised on trust and dialogue.~~ In contrast, barriers to integration seemed to exist at a systems level. They included ~~distrust, poor design and~~ fragmentation- of jurisdiction and facilities, inter-system isolation, lack of trust in and awareness of TCA systems, and inadequate infrastructure and resources for TCA service delivery.delivery~~at the systems level appears to be a barrier to integrative efforts.~~ It is a system where "little somethings" of individuals that catalyse integration are met with "nothing as such" at the systems level.

Some of our findings are not new – the experience of lack of interaction has emerged in Hollenberg's study on an integrated practice, which reported that weekly doctors' meetings included only biomedical doctors, not CAM.[19] This study also reported the "geographical dominance" of biomedical doctors in terms of location of consulting rooms, as was found in our study. A study by Broom and colleagues found tension and ~~,~~mistrust~~, and dichotomy (rational/irrational, physical/metaphysical, traditional/modern),~~ as well as ~~some~~ inconsistencies in practice~~,~~ and ~~stated~~ values related to ~~, regarding~~ biomedicine and TCAM, among Indian oncologists.[20] Such challenges were also seen in our study.

Our study also revealed some unique findings with respect to the extant literature. Chung et al, attributed low referral from biomedicine to TCAM in Hong Kong to the lack of articulated and enforced procedures of referral in an integrated medical establishment.[21] In the Indian case, it appears that the vagueness of process both allows ad hoc interactions and referrals based on personal rapport and at the same time discourages the kind of predictable, routine interactions that would allow such rapport to be built. Speaking of integration of Sowa-Rigpa in Bhutan since

For peer review only

For peer review only - http://bmjopen.bmj.com/site/about/guidelines.xhtml

Experiences and meanings of integration

1967, Wangchuk and colleagues suggest that there are managerial lessons offered by the juxtaposition and collaboration of conceptually distinct systems within a single administrative and policy unit, such as a ministry.[22]In effect, as they point out, services may not be co-located, but their administration necessarily will should be. One could argue that India's case is different – whether in facilities or administratively, it is not just two systems, but more like eight (across AYUSH systems), that are to be integrated, introducing internal hierarchies and complexities that are unique to the countrycountryand interlinked.

In the 1990s and early 2000s, it was argued that integration is about a "battle between two scientific truths," [23]or that the CAM field creates two tendencies: "uninformed skeptics who don't believe in anything, and uncritical enthusiasts who don't care about data."[24] Analysis of service delivery in India over a decade later suggests that there are multiple battles being fought – epistemological, logistical, ethical, and operational across systems, with (re)conciliatory intercession, at times, of individuals.

How can such intercessions be encouraged, catalyzed even? We offer a few suggestions for activities in the Indian case that leverage the individual facilitators of integration to fill systemic gaps (see Table 2). These strategies are based on the aforementioned findings in particular states; their 'translate-ability' to other states would have to be examined.

For one, improved documentation of clinical cases across systems could be undertaken and shared. We noted that those AYUSH practitioners who were documenting their practices had greater stature, opportunities and topics for interaction with peers. Drawing upon personal initiative and creating experiences of interaction, this could help raise the stature of TCA practice, while also reducing isolation and lack of awareness. State health departments could create routine opportunities for interaction and collaboration across systems, and within facilities. In Delhi, polio immunization has served as an integrative platform for many practitioners to work together and develop trust and ties. Within facilities, joint staff meetings

For peer review only - http://bmjopen.bmj.com/site/about/guidelines.xhtml

~~Experiences and meanings of integration~~

may serve a similar purpose. Authorities may also consider rewarding individual initiatives for integration (through challenge grants or institutional recognition) - these could be designed to address system-level barriers to integration. Systems-integration could also be rewarded, through joint or synergistically achieved targets for referrals, or number of patients cared for using complementary or adjuvant therapies. As of now, those reporting cross-referrals only know of each other; if targets were set, there would be greater incentives for and attention to conditions and protocols for cross-referral. Many practitioners we spoke to suggested that guidelines for collaboration (including cross-referral) be created. We feel this itself could be a starting point of collaboration amongst TCA providers and with allopathic providers. In each state, the feasibility of each of these strategies would have to be determined, and given due attention through the exertions of powerful stakeholders with political will, who at various points, may find themselves battling each other over policies or power.

For peer review only

Conclusion

Battles occur between armies, while acts of diplomacy involve intricate latticework relationships individuals with overlapping needs and interests. Our research across three very different Indian states – Kerala, Meghalaya and Delhi - suggests that strategies that attempt to make the health systems ~~isomorphic or~~ receptive to individual integrative efforts may facilitate integration across systems, creating opportunities for greater collaboration, and trust. ~~The~~ We have proposed strategies to this end, which must in turn be additionally ~~will accordingly need to be individually~~ tailored to~~and carefully devised~~to each state context, so that the health system exists in a vibrant but also coherent plurality of ~~the system is both more receptive to and reflective of, integrative~~ human agency.

For peer review only - http://bmjopen.bmj.com/site/about/guidelines.xhtml

~~Experiences and meanings of integration~~

Tables

Table 1. Summary of Findings

	Factors at Individual/Interpersonal Level		Factors at Group/System Level
FACILITATORS	A) **Collegiality** between practitioners within facilities<br>B) **Stature** of TCA doctors<br>C) **Personal initiative** of TCA doctors<br>D) **Personal experience** of allopaths<br>E) **Political will** of senior health system actors	BARRIERS	A) **Fragmentation** of jurisdiction and facilities<br>B) Inter-system **isolation** and lack of communication<br>C) Lack of **trust and awareness** of TCA systems<br>D) **Inadequate infrastructure and resources** for TCA service delivery

For peer review only - http://bmjopen.bmj.com/site/about/guidelines.xhtml

~~Experiences and meanings of integration~~

Table 2. Recommendations to promote/address Integration, responding to findings

	FACILITATORS				BARRIERS			
**Strategies to promote TCA integration for essential health services delivery, based on our findings**	**Collegiality**	**Stature**	**Personal Initiative**	**Personal Experience**	**Fragmentation**	**Isolation**	**Lack of trust/ awareness**	**Inadequate infrastructure/ resources**
**High level political will required for all strategies**								
Case documentation and sharing across systems, and in the academic literature		+	+	+		+	+	
Routine opportunities for interaction and collaboration across systems (eg. health camps, health promotion drives)	+			+	+	+	+	+
Routine opportunities for interaction within co-located facilities (eg. staff meetings)	+			+	+	+	+	+
Rewards for integrative initiative of individuals (eg. challenge grants or institutional recognition)	+	+	+	+				
Rewards for integrative initiative at systems or facility level (eg. Joint targets like no of monthly referrals, no of cases jointly resolved)		+		+	+	+	+	+
Guidelines for collaboration (criteria and conditions for cross-referral, jointly developed by practitioners, non-clinical aspects of work together, including health promotion and managerial duties)				+	+	+	+	+

For peer review only

~~Experiences and meanings of integration~~

References

[1] World Health Organization. WHO congress on traditional medicine 2008: Beijing declaration. WHO,2011.

2 *Resolution of the 62nd World Health Assembly on Traditional Medicine 2009*. EB124.R9. Geneva: WHA; 2009.

3 Bodeker G, Kronenberg F. A public health agenda for traditional, complementary, and alternative medicine. *Am J Public Health* 2002;92:1582–91.

4 Sheikh K, Nambiar D. Government policies for traditional, complementary and alternative medical services in India: from assimilation to integration? *Natl Med J India* 2011;24:245–6.

[5] McLeod H, Chung VCH. The Integration of Traditional and Complementary Medicine in Health Systems. *Presented at the Joint IACA, IAAHS, and PBSS Colloquium*. Hongkong. 2012. http://www.actuaries.org/HongKong2012/Presentations/TBR11_McLeod.pdf (accessed 9 Sep 2013).

[6] Quaiser N. Essentialising the body. Locating medical domination and resistance. *The Newsletter*. Autumn 2013. http://www.iias.nl/the-newsletter/article/essentialising-body-locating-medical-domination-and-resistance (accessed 21 Jan 2013).

[7] Khan S. Systems of medicine and nationalist discourse in India: Towards "new horizons" in medical anthropology and history. *SocSci Med*. 2006;62: 2786-97.

[8] World Health Organization. WHO Traditional Medicine Strategy 2002 – 2005. WHO,2002.

[9] Lohokare M, Davar BV. The Community Role of Indigenous Healers. In: Sheikh K, George A (eds.) *Health Providers in India: On the Frontlines of Change*. New Delhi: Routledge; 2011. p161–81.

For peer review only - http://bmjopen.bmj.com/site/about/guidelines.xhtml

---

[10] Department of Health and Family Welfare. *Mainstreaming AYUSH under NRHM.* http://203.193.146.66/hfw/PDF/ayus.pdf (accessed 31 Mar 2011).

[11] Department of AYUSH. *AYUSH under NRHM.* http://indianmedicine.nic.in/writereaddata/linkimages/4468616884-AYUSH%20under%20NRHM.pdf (accessed 5 Sep 2013).

[12] Unnikrishnan PM, Kumar LHP, Shankar D. Traditional Orthopaedic Practitioners' Place in Contemporary India: A Case Study from Southern India. In: Sheikh K, George A (eds.) *Health Providers in India: On the Frontlines of Change*. New Delhi: Routledge; 2011. p182–99.

[13] Lakshmi JK. Less equal than others? Experiences of AYUSH medical officers in primary health centres in Andhra Pradesh. *Indian J Med Ethics* 2012;9:18–21.

[14] Priya R, Shweta AS. *Status and Role of AYUSH and Local Health Traditions: Under the National Rural Health Mission, Report of a Study*. National Health Systems Resource Centre. 2010.

[15] Barrett S, Fudge C. *Policy and action: essays on the implementation of public policy*. London: Methuen; 1981.

[16] Walt G, Gilson L. Reforming the health sector in developing countries: the central role of policy analysis. *Health Policy Plan* 1994;9:353–70.

[17]Yanow D. *Conducting Interpretive Policy Analysis*. Newbury Park,CA: Sage; 2000.

[18] Ritchie J, Spencer L. Qualitative Data Analysis for Applied Policy Research. In *Analyzing qualitative data*. London: Routledge;1994. p173-94.

For peer review only - http://bmjopen.bmj.com/site/about/guidelines.xhtml

Experiences and meanings of integration

[19] Hollenberg D. Uncharted ground: Patterns of professional interaction among complementary/alternative and biomedical practitioners in integrative health care settings. *Soc Sci Med* 2006;62:731-44.

[20] Broom A, Doron A, Tovey P. The inequalities of medical pluralism: Hierarchies of health, the politics of tradition and the economies of care in Indian oncology. *Soc Sci Med* 2009;69:698–706.

[21] Chung VC, Hillier S, Lau CH, Wong S, Yeoh EK, Griffiths SM. Referral to and attitude towards traditional Chinese medicine amongst western medical doctors in postcolonial Hong Kong. *Soc Sci Med* 2011;72:247-55.

[22] Wangchuk P, Wangchuk D, Aagaard-Hansen J. Traditional Bhutanese medicine (gSo-BA Rig-PA): an integrated part of the formal health care services. *Southeast Asian J Trop Med Public Health* 2007;38:161–7.

[23] Bloom H. *The Lucifer Principle. A Scientific Expedition into the Forces of History*. New York: The Atlantic Monthly Press;1995.

[24] Northridge ME, Mack Jr R. Integrating ethnomedicine into public health. *Am J Public Health* 2002;92:1561-61.

For peer review only - http://bmjopen.bmj.com/site/about/guidelines.xhtml

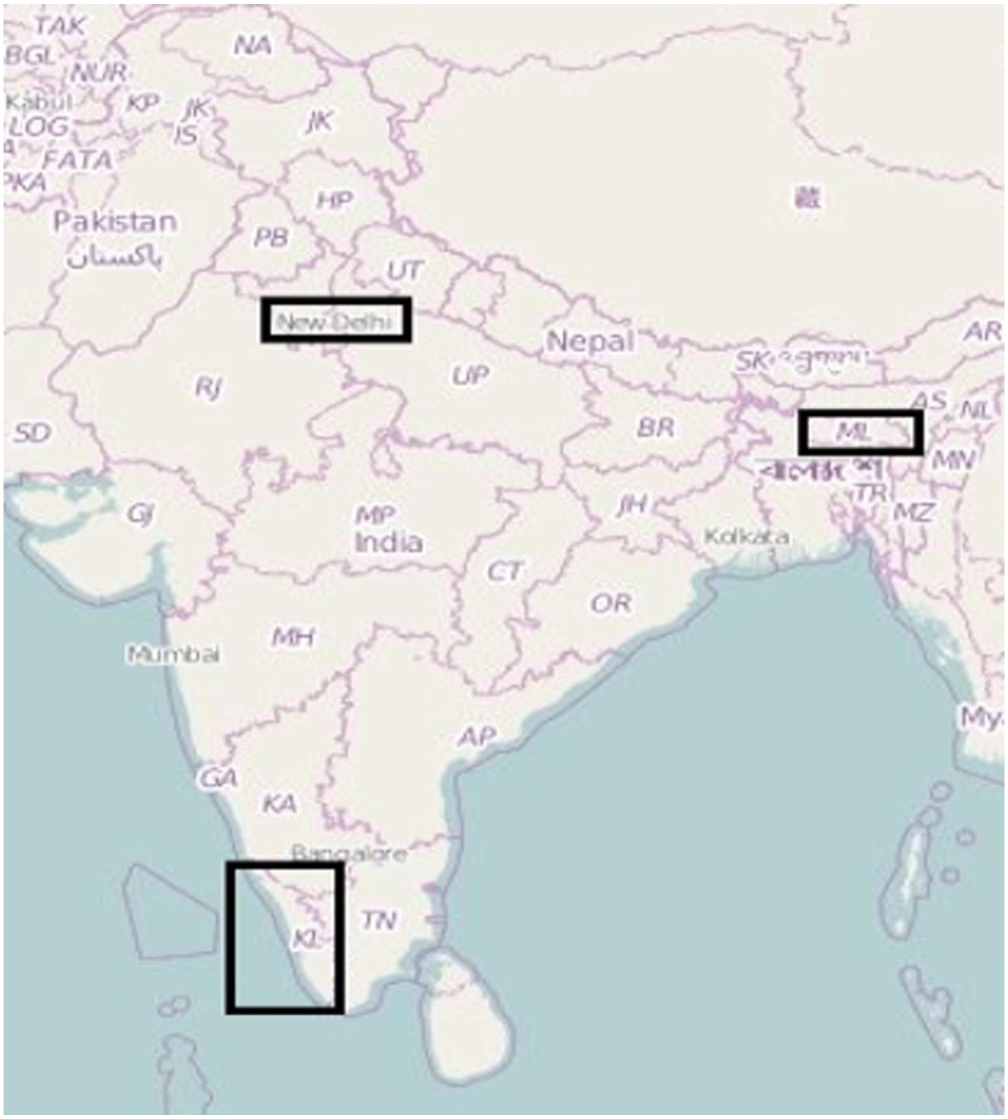

Figure 1. Location of states in India where fieldwork was conducted (New Delhi, Meghalaya (ML), and Kerala (KL))
251x279mm (300 x 300 DPI)

TOPIC GUIDE: KEY INFORMANTS

(Policy elites, representatives of TCAM and allopathic associations, CBO representatives, representatives of technical organizations)

1. Reasons for involving TCAM providers in essential health services (AYUSH and non-AYUSH)
2. Roles of TCAM providers in health services (AYUSH and non-AYUSH)
3. Existing policies and strategies for TCAM integration (AYUSH and non-AYUSH)
4. Status and extent of implementation of above policies and strategies
5. Obstacles to implementation at different levels
6. Social and cultural contexts and factors promoting and impeding integration
7. Position and role(s) of respective organization in promoting / facilitating / opposing TCAM integration

TOPIC GUIDE: HEALTH SYSTEMS ACTORS

(Public sector health planners, administrators)

1. Personal designation and role within the organization/department
2. Role and functions of organization/department
3. Designated functions of the organization/department in involving TCAM providers in service delivery
4. Organizational arrangements for performing each of these functions
5. Experiences of executing each of these functions (probe: explanations)
6. Shortfalls and obstacles in executing each function (probe: explanations)
7. Interactions with other organizations / departments in process of involving TCAM
8. Reasons for involving TCAM providers in health care system (AYUSH and non-AYUSH)
9. Roles of TCAM providers in health care system (AYUSH and non-AYUSH)
10. How is people's health care access affected as a result of TCAM involvement?
11. How is the quality of care provided by TCAM providers affected by their involvement?
12. How is the development of TCAM systems of medicine affected by their involvement?
13. Perceptions about value and utility of TCAM systems of medicine (AYUSH and non-AYUSH)
14. Opportunities to strengthen role of organization/department in working with TCAM

TOPIC GUIDE: TCAM

(TCAM practitioners working in public sector health services)

1. Your role in health care system (AYUSH and non-AYUSH)
2. Experiences of interface with administration (probe: explanations)
3. Experiences of interface with facility support staff (probe: explanations)
4. Experiences of interface with users of care and community (probe: explanations)
5. Experiences of working with allopathic providers
6. Experiences demonstrating benefits and advantages (probe: explanations)
7. Experiences demonstrating detriments and disadvantages (probe: explanations)
8. Experiences of interface with other TCAM providers [A,U,H and non-AYUSH] (probe: explanations)
9. How has the quality of care you provide been affected by involvement in health services?
10. How is the development of TCAM systems of medicine affected by involvement in health services?

TOPIC GUIDE: ALLOPATHIC DOCTORS

(Allopathic doctors working with TCAM practitioners)

1. Reasons for involving TCAM providers in health care system (AYUSH and non-AYUSH)
2. Roles of TCAM providers in health care system (AYUSH and non-AYUSH)
3. Experiences of working with TCAM providers
4. Impact of involvement of TCAM providers on facility performance (probe: explanations)
5. Experiences demonstrating benefits and advantages (probe: explanations)
6. Experiences demonstrating detriments and disadvantages (probe: explanations)
7. How is people's health care access affected as a result of TCAM involvement?
8. How is the quality of care provided by TCAM providers affected by their involvement?
9. How is the development of TCAM systems of medicine affected by their involvement?
10. Perceptions about value and utility of TCAM systems of medicine (AYUSH and non-AYUSH)

TOPIC GUIDE: COMMUNITY REPRESENTATIVES

(CBO, Consumer, MBP, PRI representatives)

1. Experience of receiving care from TCAM providers
2. Experience of receiving care from Allopathic providers
3. Experience of receiving care in co-located facilities following integration
4. How is utilization of health services affected as a result of TCAM involvement? Why?
5. Has there been any change in the quality of care in health facilities, following involvement of TCAM providers? How so?
6. Perceptions about value and utility of TCAM systems of medicine (AYUSH and non-

For peer review only

BMJ Open

Experiences and meanings of integration of TCAM (Traditional, Complementary and Alternative Medical) providers in three Indian states: Results from a cross-sectional, qualitative implementation research study

Journal:	*BMJ Open*
Manuscript ID:	bmjopen-2014-005203.R2
Article Type:	Research
Date Submitted by the Author:	02-Sep-2014
Complete List of Authors:	Nambiar, Devaki; Public Health Foundation of India,<br>Narayan, Venkatesh; All India Institute of Medical Sciences,<br>Lakshmi, JK; Indian Institute of Public Health - Hyderabad,<br>Porter, John; London School of Hygiene & Tropical Medicine, Department of Clinical Research<br>Sathyanarayana, TN; Indian Institute of Public Health-Bangalore Campus, Health Policy and Management<br>Sheikh, Kabir; Public Health Foundation of India,
**Primary Subject Heading**:	Health services research
Secondary Subject Heading:	Global health, Qualitative research
Keywords:	COMPLEMENTARY MEDICINE, International health services < HEALTH SERVICES ADMINISTRATION & MANAGEMENT, QUALITATIVE RESEARCH

SCHOLARONE™
Manuscripts

For peer review only

For peer review only - http://bmjopen.bmj.com/site/about/guidelines.xhtml

Experiences and meanings of integration

Running Head: Experiences and meanings of integration

Title: Experiences and meanings of integration of TCAM (Traditional, Complementary and Alternative Medical) providers in three Indian states: Results from a cross-sectional, qualitative implementation research study

Authors: D Nambiar,[1] VV Narayan,[2] JK Lakshmi,[3] JDH Porter,[4] TN Sathyanarayana,[3] K Sheikh[q]

1 Public Health Foundation of India, New Delhi, India

2 All India Institute of Medical Sciences, New Delhi, India

3 Indian Institute of Public Health, Hyderabad, India

4 London School of Hygiene and Tropical Medicine, London, United Kingdom

Corresponding Author Information:

Devaki Nambiar, PhD

Research Scientist

Public Health Foundation of India

ISID, 4 Institutional Area

Vasant Kunj 110067

Keywords: Alternative Medicine, Qualitative Evaluation, World Health

Word Count: 3,868

For peer review only - http://bmjopen.bmj.com/site/about/guidelines.xhtml

Abstract

Objectives: Efforts to engage Traditional Complementary and Alternative Medical (TCAM) practitioners in the public health workforce have growing relevance for India's path to universal health coverage. We used an action-centred framework to understand how policy prescriptions related to integration were being implemented in three distinct Indian states.

Setting: Health departments and district-level primary care facilities in the states of Kerala, Meghalaya, and Delhi.

Participants: In each state, two or three districts were chosen that represented variation in accessibility and distribution across TCAM providers (e.g.,small or large proportions of local health practitioners, Homoeopaths, Ayurvedic and/or Unani practitioners). Per district, two blocks or geographical units were selected. TCAM and allopathic practitioners, administrators and representatives of community at district and state levels were chosen based on publicly available records from state and municipal authorities. A total of 196 interviews were carried out: 74 in Kerala, and 61 each in Delhi and Meghalaya.

Primary and secondary outcome measures: We sought to understand experiences and meanings associated with integration across stakeholders, as well as barriers and facilitators to implementing policies related to integration of TCA providers at the systems level.

For peer review only - http://bmjopen.bmj.com/site/about/guidelines.xhtml

For peer review only

Experiences and meanings of integration

Results: We found that individual and interpersonal attributes tended to facilitate integration, while system features and processes tended to hinder it. Collegiality, recognition of stature, exercise of individual personal initiative among TCA practitioners and of personal experience of TCAM among allopaths enabled integration. The system was characterised, on the other hand, by fragmentation of jurisdiction and facilities, inter-system isolation, lack of trust in and awareness of TCA systems, and inadequate infrastructure and resources for TCA service delivery.

Conclusions: State-tailored strategies that routinise interaction, reward individual and system-level individual integrative efforts, fostered by high level political will are recommended.

For peer review only

For peer review only - http://bmjopen.bmj.com/site/about/guidelines.xhtml

Strengths

* Multi-sited qualitative study drawing on meanings and experiences across patients, providers, and health systems administrators

* Implementation research using rigorously applied interpretive policy analysis methods

* Linked to India's path to Universal Health Coverage

Limitations

* Cross-sectional study, so other than self-report of historical changes, we were not able to chart or map changed views or experiences of participants in vivo

* Focus on the public service delivery sector, even as a great deal of health-seeking takes place in the private sector, with the assumption that public sector strengthening is highly desirable, and possible only through focused study on it

For peer review only

For peer review only - http://bmjopen.bmj.com/site/about/guidelines.xhtml

Introduction

The 1978 Alma Ata declaration called for traditional medicine treatments and practices to be "preserved, promoted and communicated widely and appropriately based on the circumstances in each country." Thirty years later, the 2008 Beijing Declaration on Traditional Medicine called for integration of providers into national health systems, recommending systems of qualification, accreditation, regulation and communication (with allopathic providers).[1] These features of the Beijing Declaration were echoed at the 62nd World Health Assembly in 2009, putting out a call to action to United Nations member states to move forward with their plans for integration.[2] The global positioning of Traditional, Complementary, and Alternative Medicine (TCAM) has issued from and tends to imply a central focus on clinical and experimental medicine,[3] yet, recent calls for health systems integration, draw attention to features like education, accreditation, regulation, and health services provision, and the TCAM health workforce itself.

In earlier work, we have identified three broad trends of integration as it relates to TCA providers: self-regulation with governmental linkage, government regulation and provisioning, and hybrid/parallel models.[4] This links roughly to the WHO nosology, where three models are identified: "tolerant" systems where the national health care system is based entirely on biomedicine but some TCAM practices are legally permissible, "inclusive" systems where TCAM is recognised but not fully integrated into all aspects of healthcare, and "integrative," where TCAM is officially recognised in national drug policy, providers and products are registered and regulated, therapies are widely available and covered under insurance schemes, research and education are widely accessible.[5]

The situation on the ground in India, hybrid in our view, seems in parts to reflect tendencies across WHO categories. The dominance of biomedicine appears to be a critical feature of India's postcolonial health system, even as pre-independence, the TCAM practitioner community had

For peer review only - http://bmjopen.bmj.com/site/about/guidelines.xhtml

played a major role in resisting colonial domination in the practice of (bio)medicine.[6] In part as a response to the reliance on allopathy throughout modern Indian history, there have been strong arguments in favour of the critical role that non-mainstream practitioners play in offering accessible, affordable, and socially acceptable health services to populations.**Error! Bookmark not defined.**[,7,8] A study in Maharashtra reported that the situation of traditional healing as a community function through shared explanatory frameworks across provider and patient are explicitly unlike typical doctor-patient relationships.[9]

In India, one can also find a larger integrative framework, one that mandates "mainstreaming" of codified TCAM in India, collectively referred to as AYUSH, an acronym for Ayurveda, Yoga & Naturopathy, Unani, Siddha, Sowa-Rigpa, and Homoeopathy. The National Rural Health Mission (NRHM), launched in 2005 to fortify public health in rural India, took a particular interest in integrating AYUSH practitioners through facilitation of specialised AYUSH practice, integration of AYUSH practitioners in national health programmes, incorporation of AYUSH modalities in primary health care, strengthening the governance of AYUSH practice, support for AYUSH education, establishment of laboratories and research facilities for AYUSH, and providing infrastructural support.[10] Human resource-focused strategies included contractual appointment of AYUSH doctors in Community and Primary Health Centres, appointment of paramedics, compounders, data assistants, and managers to support AYUSH practice, establishment of specialised therapy centres for AYUSH providers, inclusion of AYUSH doctors in national disease control programmes; and incorporation of AYUSH drugs into community health workers' primary health care kits. A recent report from the AYUSH department reports that NRHM has established AYUSH facilities in co-location with health facilities in many Indian states (notably, not in Kerala, where the stand-alone AYUSH facility is the chosen norm).[11] As of 2012, more than three quarters of India's district hospitals, over half of its Community Health

For peer review only - http://bmjopen.bmj.com/site/about/guidelines.xhtml

Experiences and meanings of integration

Centres and over a third of India's Primary Health Centres have AYUSH co-location, serving about 1.77 million, 3.3 million, and 100,000 rural Indians, respectively.[11]

And yet, even this integration framework has at most an "inclusive" character. This is reflected in findings like "official neglect" of traditional orthopaedic practitioners who have no registration, uniformity in inter-state regulation, or institutionalized medical training.[12] AYUSH doctors contracted to Medical Officer posts in Primary Health Centres (PHCs) in the southern Indian state of Andhra Pradesh report numerous lacunae in the implementation of the mainstreaming initiatives in the National Rural Health Mission (NRHM):[13] job perquisites are not indicated, no benefits or allowances provided for health, housing or education, and compensation packages are much lower than those of allopathic doctors. Support for AYUSH practice is also inadequate (lack of infrastructure, trained assistants, and drug supply) and unethical practices have also been reported (documenting attendance of absentees, and non-cooperation from non-AYUSH personnel). Evidence from NRHM suggests that reshuffled AYUSH providers practice forms of medicine beyond the scope of their training.[14] Paradoxically, moreover, some Indian states prohibit cross-system prescription, adding ethical dilemmas for TCA practitioners who serve as the only medical practitioner in resource-poor areas.[14]

At a larger scale, current practices of integration (as in NRHM) have been described as substitution and replacement; which tend to ignore the merits of TCAM and present more barriers than facilitators of integration.[7] Particularly given the strong push towards co-location and other strategies of integration as part of India's move towards Universal Health Coverage, the integration of AYUSH practitioners could result in a doubling of the health workforce. And yet, there are strong fears that such an emphasis on quantitative aspects of integration, i.e. having the right number of practitioners placed at facilities is inadequate. There is a need to critically and qualitatively appraise the government infrastructure to support TCA, identify barriers and facilitators to integration that have emerged from this rapid placement of these practitioners, and

For peer review only - http://bmjopen.bmj.com/site/about/guidelines.xhtml

how these TCA practitioners, allopathic practitioners, and health system actors are reacting and adapting to each.

Methods

This analysis draws from a larger mixed methods implementation research study aimed at understanding operational and ethical challenges in integration of TCA providers for delivery of essential health services in three Indian states. The study looked at the contents and implementation of TCA provider integration policies in 3 states and at national level examining the understanding and interpretations of integration from the perspectives of different health systems actors. These coupled with their experiences in the actual processes of integration of TCA providers were studied using qualitative interview methods to help identify systemic and ethical challenges. Based on this, the study sought to derive strategies to augment the integration of TCA providers in the delivery of essential health services.

Our study was based on action-centred frameworks[15] with a focus on policy *actors* and *processes.*[16] We have therefore sought to understand the implementation of integration policies empirically. A team of four field researchers was oriented by the principal investigator and advisor to the post-positivist paradigm of research, using Yanow's model of interpretative policy analysis, where the emphasis is equally on describing the experience of policy processes, and on elaborating the meanings actors attach to those processes.[17] The research protocol was approved by the Institutional Ethics Committee of the Public Health Foundation of India.

Our methods included semi-structured in-depth interviews (see interview guides, Appendix 1) with policymakers (N=12), administrators (N=43), TCAM practitioners (N=59), allopathic practitioners (N=37), traditional healers (N=7), as well as health workers and community representatives (N=38) in three diverse Indian states (see map, Figure 1). We undertook the study in Kerala, where a number of systems have strong historical and systemic roots (N=74),

For peer review only - http://bmjopen.bmj.com/site/about/guidelines.xhtml

Meghalaya, where local health traditions hold sway (N=61), and Delhi, where national, state, and municipal jurisdictions interface with multiple systems of medicine (N=61). Participants were selected based upon maximum variation criteria for each category. We sought to represent different schemes, levels of implementation (directorates, zonal officers), systems of medicine, types of establishments (hospital, dispensary), and years of experience.

In each state, one senior researcher, a research associate and a field researcher developed selection matrices to achieve maximum variation across each category of respondents. In each state, districts (two in Kerala, and three in Meghalaya) or municipal zones (three in Delhi) were chosen to represent variation in accessibility and distribution across TCA providers (eg. small or large proportions of local health practitioners, Homoeopaths, Ayurvedic and/or Unani practitioners). Publicly available records from state and municipal authorities were consulted in order to determine location and type of facility (co-located, stand-alone) as well as suggestions and recommendations from Key Informants. We also ensured that facilities closest to and furthest from district headquarters were chosen for interviews, to maximise variability. We would typically contact providers via cell phone, share information about the study verbally or via email, and set up a time to interview them in-person. In some cases, we would arrive during out-patient clinic hours to the chosen facility, share our participant information sheet and seek an appointment time with eligible participants. In most cases, we found that participants were keen to participate once they were aware of the nature of the study and, in some cases, the assurance of confidentiality. We had no refusals, although some allopathic practitioners had to be persuaded to participate by emphasizing that this study was not "pro-TCAM integration" per se, but merely seeking to understand state policy implementation. Interviews, ranging from 15 to 90 minutes in length were undertaken, always with prior informed consent, and separate consent to record interviews. Data were transcribed and stored in password-protected folders and each transcript was checked by investigators for accuracy and quality of transcription.

For peer review only - http://bmjopen.bmj.com/site/about/guidelines.xhtml

Experiences and meanings of integration

Textual data from transcripts of interviews as well as notes and observations of facilities and service delivery recorded during fieldwork were analysed through a combination of deductive and inductive techniques in the "framework" approach of qualitative analysis for applied policy research[18] using ATLAS.ti7 software. Themes were developed in three iterations: in the first stage, the lead researcher from each state applied *a priori* codes and closely perused transcripts to devise *emergent* codes, with the support of the Research Associate. *A priori* codes were based on our research questions, reflecting experiences, interpretations and meanings of integration. *Emergent* codes were used to describe the content or categories of these experiences, interpretations and meanings. Researchers coded 20% of each other's state datasets to ensure that codes were being applied in a similar, uniform manner. In the second stage, agreement and consolidation of emergent codes across three sites took place under the direction of the study lead; these were then applied to data from each state by its respective lead researcher. Concurrently, lead researchers developed super-codes, or *analytic* codes to group emergent codes. The study lead finalised and then indexed these codes across sites to arrive at results. Emergent and analytic code families were used to develop analyses, involving sharing of data and consultation across sites. In this paper, we focus on emergent codes related to experiences and interpretations of integration.

Results

We found that facilitators of integration emerged from individual and interpersonal relationships, while barriers were identified at the systems level (see Table 1).

Facilitators at the individual/interpersonal level

A) ***Collegiality between practitioners within facilities***

Interpersonal collegiality was reported between and across some TCA and allopathic practitioners. In Meghalaya, an allopathic medical officer noted that in some places Ayurvedic

For peer review only - http://bmjopen.bmj.com/site/about/guidelines.xhtml

and Homoeopathic doctors were collaborating closely with their allopathic colleagues, expressing an interest in learning more about allopathic practices. In the same state, an AYUSH doctor described cordial relations with the administration, such that when medicine stock-outs happened, the allopathic medical officer supplied stop-gap funds to acquire medicines.

B) ***Stature of TCA doctors***

Another aspect was the "stature" of individual practitioners. In Kerala, an Ayurvedic practitioner noted that: "Nobody can question <Name of Well Known Ayurvedic Physician from Kerala>. If he says that taking *chavanaprasham* [health paste] will lead to DNA repair, then nobody can question because they are saying with authority. They are beyond questioning. If somebody else is saying [the same thing], they will ask, where is the proof?" This was also the case with a private sector entity that had opened a branch in Delhi. Practitioners in this institution were highly reputed, involved with transnational research collaborations, and reported numerous cross-referrals from allopathic providers across the city.

C) ***Personal initiative of TCA doctors***

Across states, we heard of individual TCA practitioners exercising personal initiative to hasten improvements in infrastructure and service delivery. Following is an excerpt of an interview with an Ayurvedic doctor from a Delhi hospital: "There is a lack of storage space so the diagnosis room is being used for some storage. But I have been treating people in the Public Works Department and then it is getting resolved!" Many of the participants we spoke to in many states were familiar with each other – these personal relationships and interactions, in the absence of official or regular platforms, were the basis for interaction, cross-referral, collective planning and advocacy, and in rarer cases, collaborative research.

D) ***Personal experience of allopaths***

For peer review only - http://bmjopen.bmj.com/site/about/guidelines.xhtml

Experiences and meanings of integration

Personal experience across systems also helped built trust. In Kerala, an allopath indicated that his own mother-in-law was under Ayurvedic treatment for chronic illness and that she and others he knew were "getting good relief." He noted that Ayurveda was trustworthy based on this experience. As an Ayurvedic practitioner in Delhi put it, "if one takes a personal interest, there can be a little something. But everyone is busy in their own work. If it is done officially – like in a month, every 2nd Saturday… Then it will happen more systematically."

E) ***Political will of senior health system actors***

Systems level integration was facilitated by highly networked individuals and/or individual access to top decision-makers. One of the health system actors we interviewed had participated in high level negotiations with political leaders in the country to get the AYUSH department formed (formerly the Indian Systems of Medicine & Homoeopathy department) in 1995 – which in many ways marks a critical step in the attention given to integration in the health system. Within the state of Delhi, furthermore, it was the demand articulated by city councillors and ward leaders that resulted in the construction of dispensaries and AYUSH wards in hospitals, so much so that this was considered a norm.

Barriers at the systems level

A) ***Fragmentation of jurisdictions and facilities***

It was clear that systematic integration was not widely perceived in any of the facilities or states studied. For one, all states had not a single unified system, but rather multiple systems with parallel governance apparatuses, each with their own challenges. In fact, in Delhi, integration was constrained in the system by the fragmentation of jurisdictions and facilities, but also with respect to how providers were posted at facilities. In this state, co-location did take place, but involved an individual TCA practitioner co-located at multiple sites, while multiple allopaths

For peer review only - http://bmjopen.bmj.com/site/about/guidelines.xhtml

served at a single site (the biomedical norm). Allopaths had more opportunities, in terms of sheer numbers of people, availability of space and time, to communicate with each other.

B) *Inter-system isolation and lack of communication*

Given the aforementioned lack of people, space and time, allopaths were socially isolated from, and had fewer chances to communicate with TCA providers or TCA providers with each other. In Kerala, the limitations on communication were shaped in particular by the fact that facilities tended to be stand-alone. In Meghalaya, an allopath stated, simply, "I am doing my work, and they [TCA providers] are doing theirs… that is completely asocial type, separated, segregated." There was almost no communication between local health practitioners and others – whether AYUSH or allopath simply because of a lack of systemic acknowledgement and legitimacy given to this workforce. A TCA provider remarked: "Very few people listen to our problem. Because, we are still, again, you know, under the general allopathic doctor, … so when we post our problem you know, hardly like, they table that problem…"

C) *Lack of trust and awareness of TCA systems*

When speaking about providers as a cadre, group or systems in general, we noted that distrust tended to be highlighted. In Meghalaya, an allopath opined "Please, if you want us to work in a normal way, you know, peacefully, just have these people removed." A similar sentiment was expressed by a senior Unani hospital practitioner in Delhi, "We can interact as a *pathy* but our basic concepts do not match. We can't help each other in any way. They are independent, we are independent." There was limited value, in the view of this practitioner, in engaging with other systems of medicine. An allopath in Kerala described at length how allopathic doctors had protested vehemently – and successfully – against a government policy of Ayurveda doctors getting house surgeon postings in the state. More junior practitioners noted that even with respect to TCAM systems: "We three [Ayurveda, Unani, and Homoeopathy] are together here, but cross-reference is very, very less…We don't know what is the strong point of Ayurveda,

For peer review only - http://bmjopen.bmj.com/site/about/guidelines.xhtml

Unani. Allopath will not know the strong point of Homoeopathy, Ayurveda. They just say 'skin!' – that's all they know!"

*D) **Inadequate infrastructure and resources for TCA service delivery***

Opportunities to interact were further constrained by the system design of service delivery. We observed in many dispensaries and hospitals in Delhi that non-allopathic practitioners were assigned rooms on the top floor of the facility, while allopaths were allocated multiple rooms on the ground floor (Fieldnotes June 11th, 20th, 21st, 22nd, and 27th 2012). And, most commonly, the kinds of cases that they were handling included orthopaedic ailments, and other conditions (motor, neurological, gastric) that constrained mobility and created a very real barrier of access to care within a health care facility for patients. Practitioners therefore spend much of their time responding to these inadequacies.

There were also shortcomings in the design of diagnostic services, and inadequacy of human resources. Both Homoeopathic and Ayurvedic practitioners in Kerala noted the recourse to outsourcing diagnostic investigations because of the lack of facilities in their institutions. Further, there was reliance upon contractual recruitment of human resources to address shortages, which affected the stability and reliability of service delivery, in their view. When we asked an administrator of one of Delhi's newest, state-of-the-art Ayurvedic facilities what kind of coordination occurred across departments as part of the hospital's functioning, he shrugged and replied, "Nothing as such!"

Discussion

Most striking in our findings is the emergence of individual experiences and interpretations as enablers or facilitators of integration, in the form of collegiality, recognition of stature, exercise of personal initiative among TCA practitioners and of personal experience of TCAM among allopaths. In contrast, barriers to integration seemed to exist at a systems level. They included

For peer review only - http://bmjopen.bmj.com/site/about/guidelines.xhtml

fragmentation of jurisdiction and facilities, inter-system isolation, lack of trust in and awareness of TCA systems, and inadequate infrastructure and resources for TCA service delivery. It is a system where "little somethings" of individuals that catalyse integration are met with "nothing as such" at the systems level.

Some of our findings are not new – the experience of lack of interaction has emerged in Hollenberg's study on an integrated practice, which reported that weekly doctors' meetings included only biomedical doctors, not CAM.[19] This study also reported the "geographical dominance" of biomedical doctors in terms of location of consulting rooms, as was found in our study. A study by Broom and colleagues found tension and mistrust, as well as inconsistencies in practice and values related to biomedicine and TCAM, among Indian oncologists.[20] Such challenges were also seen in our study.

Our study also revealed some unique findings with respect to the extant literature. Chung et al, attributed low referral from biomedicine to TCAM in Hong Kong to the lack of articulated and enforced procedures of referral in an integrated medical establishment.[21] In the Indian case, it appears that the vagueness of process both allows ad hoc interactions and referrals based on personal rapport and at the same time discourages the kind of predictable, routine interactions that would allow such rapport to be built. Speaking of integration of Sowa-Rigpa in Bhutan since 1967, Wangchuk and colleagues suggest that there are managerial lessons offered by the juxtaposition and collaboration of conceptually distinct systems within a single administrative and policy unit, such as a ministry.[22] In effect, as they point out, services may not be co-located, but their administration necessarily should be. One could argue that India's case is different – whether in facilities or administratively, it is not just two systems, but more like eight (across AYUSH systems), that are to be integrated, introducing internal hierarchies and complexities that are unique to the country.

For peer review only - http://bmjopen.bmj.com/site/about/guidelines.xhtml

Experiences and meanings of integration

In the 1990s and early 2000s, it was argued that integration is about a "battle between two scientific truths," [23] or that the CAM field creates two tendencies: "uninformed skeptics who don't believe in anything, and uncritical enthusiasts who don't care about data."[24] Analysis of service delivery in India over a decade later suggests that there are multiple battles being fought – epistemological, logistical, ethical, and operational across systems, with (re)conciliatory intercession, at times, of individuals.

How can such intercessions be encouraged, catalyzed even? We offer a few suggestions for activities in the Indian case that leverage the individual facilitators of integration to fill systemic gaps (see Table 2). These strategies are based on the aforementioned findings in particular states; their 'translate-ability' to other states would have to be examined.

For one, improved documentation of clinical cases across systems could be undertaken and shared. We noted that those AYUSH practitioners who were documenting their practices had greater stature, opportunities and topics for interaction with peers. Drawing upon personal initiative and creating experiences of interaction, this could help raise the stature of TCA practice, while also reducing isolation and lack of awareness. State health departments could create routine opportunities for interaction and collaboration across systems, and within facilities. In Delhi, polio immunization has served as an integrative platform for many practitioners to work together and develop trust and ties. Within facilities, joint staff meetings may serve a similar purpose. Authorities may also consider rewarding individual initiatives for integration (through challenge grants or institutional recognition) - these could be designed to address systems-level barriers to integration. Systems-integration could also be rewarded, through joint or synergistically achieved targets for referrals, or number of patients cared for using complementary or adjuvant therapies. As of now, those reporting cross-referrals only know of each other; if targets were set, there would be greater incentives for and attention to conditions and protocols for cross-referral. Many practitioners we spoke to suggested that

For peer review only - http://bmjopen.bmj.com/site/about/guidelines.xhtml

guidelines for collaboration (including cross-referral) be created. We feel this itself could be a starting point of collaboration amongst TCA providers and with allopathic providers. In each state, the feasibility of each of these strategies would have to be determined, and given due attention through the exertions of powerful stakeholders with political will, who at various points, may find themselves battling each other over policies or power.

Conclusion

Battles occur between armies, while acts of diplomacy involve intricate latticework relationships among individuals with overlapping needs and interests. Our research across three very different Indian states – Kerala, Meghalaya and Delhi – suggests that strategies that attempt to make the health systems receptive to individual integrative efforts may facilitate integration across systems, creating opportunities for greater collaboration, and trust. We have proposed strategies to this end, which must in turn be additionally tailored to each state context, so that the health system exists in a vibrant but also coherent plurality of human agency.

For peer review only

For peer review only - http://bmjopen.bmj.com/site/about/guidelines.xhtml

Tables

Table 1. Summary of Findings

	Factors at Individual/Interpersonal Level		Factors at Group/System Level
FACILITATORS	A) **Collegiality** between practitioners within facilities<br>B) **Stature** of TCA doctors<br>C) **Personal initiative** of TCA doctors<br>D) **Personal experience** of allopaths<br>E) **Political will** of senior health system actors	BARRIERS	A) **Fragmentation** of jurisdiction and facilities<br>B) Inter-system **isolation** and lack of communication<br>C) Lack of **trust and awareness** of TCA systems<br>D) **Inadequate infrastructure and resources** for TCA service delivery

For peer review only - http://bmjopen.bmj.com/site/about/guidelines.xhtml

Table 2. Strategies to increase facilitators and decrease barriers to Integration, corresponding with study findings

**Strategies that may enhance TCA integration for essential health services delivery, based on our findings**	Strategies that promote **FACILITATORS**				Strategies that remove **BARRIERS**			
	Collegiality	Stature	Personal Initiative	Personal Experience	Fragmentation	Isolation	Lack of trust/ awareness	Inadequate infrastructure/ resources
**High level political will required for all strategies**								
Case documentation and sharing across systems, and in the academic literature		✓	✓	✓		✓	✓	
Routine opportunities for interaction and collaboration across systems (eg. health camps, health promotion drives)	✓			✓	✓	✓	✓	✓
Routine opportunities for interaction within co-located facilities (eg. staff meetings)	✓			✓	✓	✓	✓	✓
Rewards for integrative initiative of individuals (eg. challenge grants or institutional recognition)	✓	✓	✓	✓				
Rewards for integrative initiative at systems or facility level (eg. joint targets like no. of monthly referrals, no. of cases jointly resolved)		✓		✓	✓	✓	✓	✓
Guidelines for collaboration (criteria and conditions for cross-referral, jointly developed by practitioners, non-clinical aspects of work together, including health promotion and managerial duties)				✓	✓	✓	✓	✓

For peer review only

For peer review only - http://bmjopen.bmj.com/site/about/guidelines.xhtml

Acknowledgements

This research was supported by a Wellcome Trust Capacity Strengthening Strategic Award to the Public Health Foundation of India and a consortium of UK universities. We are grateful for the field support of Kaveri Mayra, Candida Thangkhiew, Bobylin Nadon, Darisuk Kharlyngdoh, Ivanhoe Marak, as well as Sabitha Chandran, and the guidance of Dr. Sandra Albert.

Contributorship statement

Kabir Sheikh and John DH Porter made substantial contributions to the conception or design of the work; while Devaki Nambiar, Venkatesh Narayan, JK Lakshmi, and TN Sathyanarayana made substantial contributions to the acquisition of data. All authors substantially contributed to the analysis, and interpretation of data for the work. With Devaki Nambiar playing a lead, coordinating role in drafting the work, all authors revised it critically for important intellectual content, giving final approval of the version to be published. Further, all authors agree to be accountable for all aspects of the work in ensuring that questions related to the accuracy or integrity of any part of the work are appropriately investigated and resolved.

Competing Interests

None declared.

For peer review only - http://bmjopen.bmj.com/site/about/guidelines.xhtml

For peer review only

Funding

This research was supported by a Wellcome Trust Capacity Strengthening Strategic Award to the Public Health Foundation of India and a consortium of UK universities.

Data sharing

No additional data available.

For peer review only

References

[1] World Health Organization. WHO congress on traditional medicine 2008: Beijing declaration. WHO,2011.

2 *Resolution of the 62nd World Health Assembly on Traditional Medicine 2009*. EB124.R9. Geneva: WHA; 2009.

3 Bodeker G, Kronenberg F. A public health agenda for traditional, complementary, and alternative medicine. *Am J Public Health* 2002;92:1582–91.

4 Sheikh K, Nambiar D. Government policies for traditional, complementary and alternative medical services in India: from assimilation to integration? *Natl Med J India* 2011;24:245–6.

[5] McLeod H, Chung VCH. The Integration of Traditional and Complementary Medicine in Health Systems. *Presented at the Joint IACA, IAAHS, and PBSS Colloquium*. Hongkong. 2012. http://www.actuaries.org/HongKong2012/Presentations/TBR11_McLeod.pdf (accessed 9 Sep 2013).

[6] Quaiser N. Essentialising the body. Locating medical domination and resistance. *The Newsletter*. Autumn 2013. http://www.iias.nl/the-newsletter/article/essentialising-body-locating-medical-domination-and-resistance (accessed 21 Jan 2013).

[7] Khan S. Systems of medicine and nationalist discourse in India: Towards "new horizons" in medical anthropology and history. *SocSci Med*. 2006;62: 2786-97.

[8] World Health Organization. WHO Traditional Medicine Strategy 2002 – 2005. WHO,2002.

For peer review only - http://bmjopen.bmj.com/site/about/guidelines.xhtml

[9] Lohokare M, Davar BV. The Community Role of Indigenous Healers. In: Sheikh K, George A (eds.) *Health Providers in India: On the Frontlines of Change*. New Delhi: Routledge; 2011. p161–81.

[10] Department of Health and Family Welfare. *Mainstreaming AYUSH under NRHM*. http://203.193.146.66/hfw/PDF/ayus.pdf (accessed 31 Mar 2011).

[11] Department of AYUSH. *AYUSH under NRHM*. http://indianmedicine.nic.in/writereaddata/linkimages/4468616884-AYUSH%20under%20NRHM.pdf (accessed 5 Sep 2013).

[12] Unnikrishnan PM, Kumar LHP, Shankar D. Traditional Orthopaedic Practitioners' Place in Contemporary India: A Case Study from Southern India. In: Sheikh K, George A (eds.) *Health Providers in India: On the Frontlines of Change*. New Delhi: Routledge; 2011. p182–99.

[13] Lakshmi JK. Less equal than others? Experiences of AYUSH medical officers in primary health centres in Andhra Pradesh. *Indian J Med Ethics* 2012;9:18–21.

[14] Priya R, Shweta AS. *Status and Role of AYUSH and Local Health Traditions: Under the National Rural Health Mission, Report of a Study*. National Health Systems Resource Centre. 2010.

[15] Barrett S, Fudge C. *Policy and action: essays on the implementation of public policy*. London: Methuen; 1981.

[16] Walt G, Gilson L. Reforming the health sector in developing countries: the central role of policy analysis. *Health Policy Plan* 1994;9:353–70.

[17]Yanow D. *Conducting Interpretive Policy Analysis*. Newbury Park,CA: Sage; 2000.

[18] Ritchie J, Spencer L. Qualitative Data Analysis for Applied Policy Research. In *Analyzing qualitative data*. London: Routledge;1994. p173-94.

For peer review only - http://bmjopen.bmj.com/site/about/guidelines.xhtml

[19] Hollenberg D. Uncharted ground: Patterns of professional interaction among complementary/alternative and biomedical practitioners in integrative health care settings. *Soc Sci Med* 2006;62:731-44.

[20] Broom A, Doron A, Tovey P. The inequalities of medical pluralism: Hierarchies of health, the politics of tradition and the economies of care in Indian oncology. *Soc Sci Med* 2009;69:698–706.

[21] Chung VC, Hillier S, Lau CH, et al. Referral to and attitude towards traditional Chinese medicine amongst western medical doctors in postcolonial Hong Kong. *Soc Sci Med* 2011;*72*:247-55.

[22] Wangchuk P, Wangchuk D, Aagaard-Hansen J. Traditional Bhutanese medicine (gSo-BA Rig-PA): an integrated part of the formal health care services. *Southeast Asian J Trop Med Public Health* 2007;38:161–7.

[23] Bloom H. *The Lucifer Principle. A Scientific Expedition into the Forces of History*. New York: The Atlantic Monthly Press;1995.

[24] Northridge ME, Mack Jr R. Integrating ethnomedicine into public health. *Am J Public Health* 2002;92:1561-61.

For peer review only

For peer review only - http://bmjopen.bmj.com/site/about/guidelines.xhtml

Experiences and meanings of integration

Running Head: Experiences and meanings of integration

Title: Experiences and meanings of integration of TCAM (Traditional, Complementary and Alternative Medical) providers in three Indian states: Results from a cross-sectional, qualitative implementation research study

For peer review only

Authors: D Nambiar,[1] VV Narayan,[2] JK Lakshmi,[3] JDH~~J~~ Porter,[4] TN Sathyanarayana,[3] K Sheikh[q]

1 Public Health Foundation of India, New Delhi, India

2 All India Institute of Medical Sciences, New Delhi, India

3 Indian Institute of Public Health, Hyderabad, India

4 London School of Hygiene and Tropical Medicine, London, United Kingdom

Corresponding Author Information:

Devaki Nambiar, PhD

Research Scientist

Public Health Foundation of India

ISID, 4 Institutional Area

Vasant Kunj 110067

Keywords: Alternative Medicine, Qualitative Evaluation, World Health

Word Count: 3,868

1 of 21

Acknowledgements

This research was supported by a Wellcome Trust Capacity Strengthening Strategic Award to the Public Health Foundation of India and a consortium of UK universities. We are grateful for the field support of Kaveri Mayra, Candida Thangkhiew, Bobylin Nadon, Darisuk Kharlyngdoh, Ivanhoe Marak, as well as Sabitha Chandran, and the guidance of Dr. Sandra Albert.

For peer review only

Strengths

* Multi-sited qualitative study drawing on meanings and experiences across patients, providers, and health systems administrators

* Implementation research using rigorously~~rigourously~~ applied interpretive policy analysis methods

* Linked to India's path to~~on~~ Universal Health Coverage

Limitations

* Cross-sectional study, so other than self-report of historical changes, we were not able to chart or map changed views or experiences of participants in vivo~~.~~

* Focus on the public service delivery sector, even as a great deal of health-seeking takes place in the private sector, with the assumption that public sector strengthening is highly desirable, and possible only through focused study on it~~.~~

Competing Interests

None declared.

Experiences and meanings of integration

Contributorship statement

Kabir Sheikh and John DH Porter made substantial contributions to the conception or design of the work; while Devaki Nambiar, Venkatesh Narayan, JK Lakshmi, and TN Sathyanarayana made substantial contributions to the acquisition of data. All authors substantially contributed to the analysis, and interpretation of data for the work. With Devaki Nambiar playing a lead, coordinating role in drafting the work, all authors revised it critically for important intellectual content, giving final approval of the version to be published. Further, all authors agree to be accountable for all aspects of the work in ensuring that questions related to the accuracy or integrity of any part of the work are appropriately investigated and resolved.

Competing interests

None to declare.

Funding

This research was supported by a Wellcome Trust Capacity Strengthening Strategic Award to the Public Health Foundation of India and a consortium of UK universities.

Data sharing

No additional data available.

For peer review only

Introduction

The 1978 Alma Ata declaration called for traditional medicine, treatments and practices to be "preserved, promoted and communicated widely and appropriately based on the circumstances in each country." Thirty years later, the 2008 Beijing Declaration on Traditional Medicine called for integration of providers into national health systems, recommending systems of qualification, accreditation, regulation and communication (with allopathic providers).[1] These features of the Beijing Declaration were echoed at the 62nd World Health Assembly in 2009, putting out a call to action to United Nations member states to move forward with their plans for integration.[2] The global positioning of Traditional, Complementary, and Alternative Medicine (TCAM) has issued from and tends to imply a central focus on clinical and experimental medicine,[3] yet, recent calls for health systems integration, drawdrawing attention to features like education, accreditation, regulation, and health services provision, and draw attention to the TCAM health workforce itself.

In earlier work, we have identified three broad trends of integration as it relates to TCA providers: self-regulation with governmental linkage, government regulation and provisioning, and hybrid/parallel models.[4] This links roughly to the WHO nosology, where three models are identified: "tolerant" systems where the national health care system is based entirely on biomedicine but some TCAM practices are legally permissible, "inclusive" systems where TCAM is recognised but not fully integrated into all aspects of healthcare, and "integrative," where TCAM is officially recognised in national drug policy, providers and products are registered and regulated, therapies are widely available and covered under insurance schemes, research and education are widely accessible.[5]

The situation on the ground in India, hybrid in our view, seems in parts to reflect tendencies across WHO categories. The dominance of biomedicine appears to be a critical feature of India's

For peer review only - http://bmjopen.bmj.com/site/about/guidelines.xhtml

postcolonial health system, even as pre-independence, the TCAM practitioner community had played a major role in resisting colonial domination in the practice of (bio)medicine.[6] In part as a response to the reliance on allopathy throughout modern Indian history, there have been strong arguments in favour of the critical role that non-mainstream practitioners play in offering accessible, affordable, and socially acceptable health services to populations.**Error! Bookmark not defined.**[,7,8] A study in Maharashtra reported that the situation of traditional healing as a community function through shared explanatory frameworks across provider and patient are explicitly unlike typical doctor-patient relationships.[9]

In India, one can also find a larger integrative framework, one that mandates "mainstreaming" of codified TCAM in India, collectively referred to as AYUSH, an acronym for Ayurveda, Yoga & Naturopathy, Unani, Siddha, Sowa-Rigpa, and Homoeopathy. The National Rural Health Mission (NRHM), launched in 2005 to fortify public health in rural India, took a particular interest in integrating AYUSH practitioners through facilitation of specialised AYUSH practice, integration of AYUSH practitioners in national health programmes, incorporation of AYUSH modalities in primary health care, strengthening the governance of AYUSH practice, support for AYUSH education, establishment of laboratories and research facilities for AYUSH, and providing infrastructural support.[10] Human resource-focused strategies included contractual appointment of AYUSH doctors in Community and Primary Health Centres, appointment of paramedics, compounders, data assistants, and managers to support AYUSH practice, establishment of specialised therapy centres for AYUSH providers, inclusion of AYUSH doctors in national disease control programmes; and incorporation of AYUSH drugs into community health workers' primary health care kits. A recent report from the AYUSH department reports that NRHM has established AYUSH facilities in co-location with health facilities in many Indian states (notably, not in Kerala, where the stand-alone AYUSH facility is the chosen norm).[11] As of 2012, more than three quarters of India's district hospitals, over half of its Community Health

For peer review only - http://bmjopen.bmj.com/site/about/guidelines.xhtml

Experiences and meanings of integration

Centres and over a third of India's Primary Health Centres have AYUSH co-location, serving about 1.77 million, 3.3 million, and 100,000 rural Indians, respectively.[11]

And yet, even this integration framework has at most an "inclusive" character. This is reflected in findings like "official neglect" of traditional orthopaedic practitioners who have no registration, uniformity in inter-state regulation, or institutionalized medical training.[12] AYUSH doctors contracted to Medical Officer posts in Primary Health Centres (PHCs) in the southern Indian state of Andhra Pradesh report numerous lacunae in the implementation of the mainstreaming initiatives in the National Rural Health Mission (NRHM):[13] job perquisites are not indicated, no benefits or allowances provided for health, housing or education, and compensation packages are much lower than those of allopathic doctors. Support for AYUSH practice is also inadequate (lack of infrastructure, trained assistants, and drug supply) and unethical practices have also been reported (documenting attendance of absentees, and non-cooperation from non-AYUSH personnel). Evidence from NRHM suggests that reshuffled AYUSH providers practice forms of medicine beyond the scope of their training.[14] Paradoxically, moreover, some Indian states prohibit cross-system prescription, adding ethical dilemmas for TCA practitioners who serve as the only medical practitioner in resource-poor areas.[14]

At a larger scale, current practices of integration (as in NRHM) have been described as substitution and replacement; which tend to ignore the merits of TCAM and present more barriers than facilitators of integration.[7] Particularly given the strong push towards co-location and other strategies of integration as part of India's move towards Universal Health Coverage, the integration of AYUSH practitioners could result in a doubling of the health workforce. And yet, there are strong fears that such an emphasis on quantitative aspects of integration, i.e. having the right number of practitioners placed at facilities is inadequate. There is a need to critically and qualitatively appraise the government infrastructure to support TCA, identify barriers and facilitators to integration that have emerged from this rapid placement of these practitioners, and

For peer review only

Experiences and meanings of integration

how these TCA practitioners, allopathic practitioners, and health system actors are reacting and adapting to each.

Methods

This analysis draws from a larger mixed methods implementation research study aimed at understanding operational and ethical challenges in integration of TCA providers for delivery of essential health services in three Indian states. The study looked at the contents and implementation of TCA provider integration policies in 3 states and at national level examining the understanding and interpretations of integration from the perspectives of different health systems actors. These coupled with their experiences in the actual processes of integration of TCA providers were studied using qualitative interview methods to help identify systemic and ethical challenges. Based on this, the study sought to derive strategies to augment the integration of TCA providers in the delivery of essential health services.

Our study was based on action-centred frameworks[15] with a focus on policy *actors* and *processes*.[16] We have therefore sought to understand the implementation of integration policies empirically. A team of four field researchers was oriented by the principal investigator and advisor to the post-positivist paradigm of research, using Yanow's model of interpretative policy analysis, where the emphasis is equally on describing the experience of policy processes, and on elaborating the meanings actors attach to those processes.[17] The research protocol was approved by the Institutional Ethics Committee of the Public Health Foundation of India.

Our methods included semi-structured in-depth interviews (see interview guides, Appendix 1) with policymakers (N=12), administrators (N=43), TCAM practitioners, (N=59), allopathic practitioners (N=37), traditional healers (N=7), as well as health workers and community representatives (N=38) in three diverse Indian states (see map, Figure 1). We undertook the study in Kerala, where a number of systems have strong historical and systemic roots (N=74),

For peer review only

Experiences and meanings of integration

Meghalaya, where local health traditions hold sway (N=61), and Delhi, where national, state, and municipal jurisdictions interface with multiple systems of medicine (N=61).- Participants were selected based upon maximum variation criteria for each category. We sought to represent different schemes, levels of implementation (directorates, zonal officers), systems of medicine, types of establishments (hospital, dispensary), and years of experience.

In each state, one senior researcher, a research associate and a field researcher developed selection matrices to achieve maximum variation across each category of respondents. In each state, two districts (two were chosen (in Kerala, andthe case of Delhi, three in Meghalaya) or municipal zones (three in Delhi) were chosen to represent) that represented variation in accessibility and distribution across TCA providers (eg. small or large proportions of local health practitioners, Homoeopaths, Ayurvedic and/or Unani practitioners). Publicly available records from state and municipal authorities were consulted in order to determine location and type of facility (co-located, stand-alone) as well as suggestions and recommendations from Key Informants. We also ensured that facilities closest to and furthest from district headquarters were chosen for interviews, to maximise variability. We would typically contact providers via cell phone, share information about the study verbally or via email, and set up a time to interview them in-person. In some cases, we would arrive during out-patient clinic hours to the chosen facility, share our participant information sheet and seek an appointment time with eligible participants. In most cases, we found that participants were keen to participate once they were aware of the nature of the study and, in some cases, the assurance of confidentiality. We had no refusals, although some allopathic practitioners had to be persuaded to participate by emphasizing that this study was not “pro-TCAM integration” per se, but merely seeking to understand state policy implementation. Interviews, ranging from 15 to 90 minutes in length were undertaken, always with prior informed consent, and separate consent to record interviews.

For peer review only

Experiences and meanings of integration

Data were transcribed and stored in password-protected folders and each transcript was checked by investigators for accuracy and quality of transcription.

Textual data from transcripts of interviews as well as notes and observations of facilities and service delivery recorded during fieldwork were analysed through a combination of deductive and inductive techniques in the "framework" approach of qualitative analysis for applied policy research[18] using ATLAS.ti7 software. Themes were developed in three iterations: in the first stage, the lead researcher from each state applied *a priori* codes and closely perused transcripts to devise *emergent* codes, with the support of the Research Associate. *A priori* codes were based on our research questions, reflecting experiences, interpretations and meanings of integration. (eg. Tc_Ap_El_Adm refers to a TCAM providers' explanation of experience of interactions with administration in the facility or the health care system). *Emergent* codes were used to describe the content or categories of these experiences, interpretations and meanings. (eg. Em_El_IndInit refers to personal initiative as a determinant of integration). Researchers coded 20% of each other's state datasets to ensure that codes were being applied in a similar, uniform manner. In the second stage, agreement and consolidation of emergent codes across three sites took place under the direction of the study lead; these were then applied to data from each state by its respective lead researcher. Concurrently, lead researchers developed super-codes, or *analytic* codes to group emergent codes. (eg. An_Ope_Adhoc refers to adhocism in policies and practices related to integration). The study lead finalised and then indexed these codes across sites to arrive at results. Emergent and analytic code families were used to develop analyses, involving sharing of data and consultation across sites. In this paper, we focus on emergent codes related to experiences and interpretations of integration.

For peer review only - http://bmjopen.bmj.com/site/about/guidelines.xhtml

Experiences and meanings of integration

Results

We found that facilitators of integration emerged from individual and interpersonal relationships, while barriers were identified at the systems level (see Table 1).

Facilitators at the individual/interpersonal level

A) ***Collegiality between practitioners within facilities***

-Interpersonal collegiality was reported between and across some TCA and allopathic practitioners. In Meghalaya, an allopathic medical officer noted that in some places Ayurvedic and Homoeopathic doctors were collaborating closely with their allopathic~~his~~ colleagues, expressing an interest in learning more about allopathic practices. In the same state, an AYUSH doctor described cordial relations with the administration, such that when medicine stock-outs happened, the allopathic medical officer supplied stop-gap funds to acquire medicines.

B) ***Stature of TCA doctors***

Another aspect was the "stature" of individual practitioners. In Kerala, an Ayurvedic practitioner noted that: "Nobody can question <Name of Well Known Ayurvedic Physician from Kerala>. If he says that taking *chavanaprasham* [health paste] will lead to DNA repair, then nobody can question because they are saying with authority. They are beyond questioning. If somebody else is saying [the same thing],~~]~~ they will ask, where is the proof?" This was also the case with a private sector entity that had opened a branch in Delhi. Practitioners in this institution were highly reputed, involved with transnational research collaborations, and reported numerous cross-referrals from allopathic providers across the city.

C) ***Personal initiative of TCA doctors***

Across states, we heard of individual TCA practitioners exercising personal initiative to hasten improvements in infrastructure and service delivery. Following is an excerpt of an interview with

For peer review only - http://bmjopen.bmj.com/site/about/guidelines.xhtml

Experiences and meanings of integration

an Ayurvedic doctor from a Delhi hospital: "There is a lack of storage space so the diagnosis room is being used for some storage. But I have been treating people in the Public Works Department and then it is getting resolved!" Many of the participants we spoke to in many states were familiar with each other – these personal relationships and interactions, in the absence of official or regular platforms, were the basis for interaction, cross-referral, collective planning and advocacy, and in rarer cases, collaborative research.

D) ***Personal experience of allopaths***

Personal experience across systems also helped built trust. In Kerala, an allopath indicated that his own mother-in-law was under Ayurvedic treatment for chronic illness and that she and others he knew were "getting good relief." He noted that Ayurveda was trustworthy based on this experience. As an Ayurvedic practitioner in Delhi put it, "if one takes a personal interest, there can be a little something. But everyone is busy in their own work. If it is done officially – like in a month, every 2nd Saturday… ~~…~~Then it will happen more systematically."

E) ***Political will of senior health system actors***

Systems level integration was facilitated by highly networked individuals and/or individual access to top decision-makers. One of the health system actors we interviewed had participated in high level negotiations with political leaders in the country to get the AYUSH department formed (formerly the Indian Systems of Medicine & Homoeopathy department) in 1995 – which in many ways marks a critical step in the attention given to integration in the health system. Within the state of Delhi, furthermore, it was the demand articulated by city councillors and ward leaders that resulted in the construction of dispensaries and AYUSH wards in hospitals, so much so that this was considered a norm.

Barriers at the systems~~system~~ level

A) ***Fragmentation of jurisdictions and facilities***

For peer review only

Experiences and meanings of integration

It was clear that systematic integration was not widely perceived in any of the facilities or states studied. For one, all states had not a single unified system, but rather multiple systems with parallel governance apparatuses, each with their own challenges. In fact, in Delhi, integration was constrained in the system by the fragmentation of jurisdictions and facilities, but also with respect to how providers were posted at facilities. In this state, co-location did take place, but involved an individual TCA practitioner co-located at multiple sites, while multiple allopaths served at a single site (the biomedical norm). Allopaths had more opportunities, in terms of sheer numbers of people, availability of space and time, to communicate with each other.

B) ***Inter-system isolation and lack of communication***

Given the aforementioned lack of people, space and time, allopaths were socially isolated from, and had fewer chances to communicate with TCA providers or TCA providers with each other. In Kerala, the limitations on communication were shaped in particular by the fact that facilities tended to be stand-alone. In Meghalaya, an allopathAyurveda doctor stated, simply, "I am doing my work, and they [TCA providers] are doing theirs… that is completely asocial type, separated, segregated." There was almost no communication between local health practitioners and others – whether AYUSH or allopath simply because of a lack of systemic acknowledgement and legitimacy given to this workforce. A TCA provider remarked: "VeryThis doctor went on: "very few people listen to our problem. Because, we are still, again, you know, under the general allopathic doctor, … so when we post our problem you know, hardly like, they table that problem…"

C) ***Lack of trust and awareness of TCA systems***

When speaking about providers as a cadre, group or systems in general, we noted that distrust tended to be highlighted. In Meghalaya, an allopath opined "Please, if you want us to work in a normal way, you know, peacefully, just have these people removed." A similar sentiment was expressed by a senior Unani hospital practitioner in Delhi, "We can interact as a *pathy* but our

For peer review only - http://bmjopen.bmj.com/site/about/guidelines.xhtml

basic concepts do not match. We can't help each other in any way. They are independent, we are independent." There was limited value, in the view of this practitioner, in engaging with other systems of medicine. An allopath in Kerala described at length how allopathic doctors had protested vehemently – and successfully – against a government policy of Ayurveda doctors getting house surgeon postings in the state. More junior practitioners noted that even with respect to TCAM systems: "We three [Ayurveda, Unani, and Homoeopathy] are together here, but cross-reference is very, very less…We don't know what is the strong point of Ayurveda, Unani. Allopath will not know the strong point of Homoeopathy, Ayurveda. They just say 'skin!' – that's all they know!"

D) ***Inadequate infrastructure and resources for TCA service delivery***

Opportunities to interact were further constrained by the system design of service delivery. We observed in many dispensaries and hospitals in Delhi that non-allopathic practitioners were assigned rooms on the top floor of the facility, while allopaths were allocated multiple rooms on the ground floor (Fieldnotes June 11th, 20th, 21st, 22nd, and 27th 2012). And, most commonly, the kinds of cases that they were handling included orthopaedic ailments, and other conditions (motor, neurological, gastric) that constrained mobility and created a very real barrier of access to care within a health care facility for patients. Practitioners therefore spend much of their time responding to these inadequacies.

There were also shortcomings in the design of diagnostic services, and inadequacy of human resources. Both Homoeopathic and Ayurvedic practitioners in Kerala noted the recourse to outsourcing diagnostic investigations because of the lack of facilities in their institutions. Further, there was reliance upon contractual recruitment of human resources to address shortages, which affected the stability and reliability of service delivery, in their view. When we asked an administrator of one of Delhi's newest, state-of-the-art Ayurvedic facilities what kind of

For peer review only

coordination occurred across departments as part of the hospital's functioning, he shrugged and replied, "Nothing as such!"

Discussion

Most striking in our findings is the emergence of individual experiences and interpretations as enablers or facilitators of integration, in the form of collegiality, recognition of stature, exercise of personal initiative among TCA practitioners and of personal experience of TCAM among allopaths. In contrast, barriers to integration seemed to exist at a systems level. They included fragmentation of jurisdiction and facilities, inter-system isolation, lack of trust in and awareness of TCA systems, and inadequate infrastructure and resources for TCA service delivery. It is a system where "little somethings" of individuals that catalyse integration are met with "nothing as such" at the systems level.

Some of our findings are not new – the experience of lack of interaction has emerged in Hollenberg's study on an integrated practice, which reported that weekly doctors' meetings included only biomedical doctors, not CAM.[19] This study also reported the "geographical dominance" of biomedical doctors in terms of location of consulting rooms, as was found in our study. A study by Broom and colleagues found tension and mistrust, as well as inconsistencies in practice and values related to biomedicine and TCAM, among Indian oncologists.[20] Such challenges were also seen in our study.

Our study also revealed some unique findings with respect to the extant literature. Chung et al, attributed low referral from biomedicine to TCAM in Hong Kong to the lack of articulated and enforced procedures of referral in an integrated medical establishment.[21] In the Indian case, it appears that the vagueness of process both allows ad hoc interactions and referrals based on personal rapport and at the same time discourages the kind of predictable, routine interactions that would allow such rapport to be built. Speaking of integration of Sowa-Rigpa in Bhutan since

For peer review only - http://bmjopen.bmj.com/site/about/guidelines.xhtml

Experiences and meanings of integration

1967, Wangchuk and colleagues suggest that there are managerial lessons offered by the juxtaposition and collaboration of conceptually distinct systems within a single administrative and policy unit, such as a ministry.[22] In effect, as they point out, services may not be co-located, but their administration necessarily should be. One could argue that India's case is different – whether in facilities or administratively, it is not just two systems, but more like eight (across AYUSH systems), that are to be integrated, introducing internal hierarchies and complexities that are unique to the country.

In the 1990s and early 2000s, it was argued that integration is about a "battle between two scientific truths,"[23] or that the CAM field creates two tendencies: "uninformed skeptics who don't believe in anything, and uncritical enthusiasts who don't care about data."[24] Analysis of service delivery in India over a decade later suggests that there are multiple battles being fought – epistemological, logistical, ethical, and operational across systems, with (re)conciliatory intercession, at times, of individuals.

How can such intercessions be encouraged, catalyzed even? We offer a few suggestions for activities in the Indian case that leverage the individual facilitators of integration to fill systemic gaps (see Table 2). These strategies are based on the aforementioned findings in particular states; their 'translate-ability' to other states would have to be examined.

For one, improved documentation of clinical cases across systems could be undertaken and shared. We noted that those AYUSH practitioners who were documenting their practices had greater stature, opportunities and topics for interaction with peers. Drawing upon personal initiative and creating experiences of interaction, this could help raise the stature of TCA practice, while also reducing isolation and lack of awareness. State health departments could create routine opportunities for interaction and collaboration across systems, and within facilities. In Delhi, polio immunization has served as an integrative platform for many practitioners to work together and develop trust and ties. Within facilities, joint staff meetings

For peer review only - http://bmjopen.bmj.com/site/about/guidelines.xhtml

may serve a similar purpose. Authorities may also consider rewarding individual initiatives for integration (through challenge grants or institutional recognition) - these could be designed to address systemssystem-level barriers to integration. Systems-integration could also be rewarded, through joint or synergistically achieved targets for referrals, or number of patients cared for using complementary or adjuvant therapies. As of now, those reporting cross-referrals only know of each other; if targets were set, there would be greater incentives for and attention to conditions and protocols for cross-referral. Many practitioners we spoke to suggested that guidelines for collaboration (including cross-referral) be created. We feel this itself could be a starting point of collaboration amongst TCA providers and with allopathic providers. In each state, the feasibility of each of these strategies would have to be determined, and given due attention through the exertions of powerful stakeholders with political will, who at various points, may find themselves battling each other over policies or power.

Conclusion

Battles occur between armies, while acts of diplomacy involve intricate latticework relationships among individuals with overlapping needs and interests. Our research across three very different Indian states – Kerala, Meghalaya and Delhi –- suggests that strategies that attempt to make the health systems receptive to individual integrative efforts may facilitate integration across systems, creating opportunities for greater collaboration, and trust. We have proposed strategies to this end, which must in turn be additionally tailored to each state context, so that the health system exists in a vibrant but also coherent plurality of -human agency.

For peer review only

Tables

Table 1. Summary of Findings

	Factors at Individual/Interpersonal Level		Factors at Group/System Level
**FACILITATORS**	A) **Collegiality** between practitioners within facilities<br>B) **Stature** of TCA doctors<br>C) **Personal initiative** of TCA doctors<br>D) **Personal experience** of allopaths<br>E) **Political will** of senior health system actors	**BARRIERS**	A) **Fragmentation** of jurisdiction and facilities<br>B) Inter-system **isolation** and lack of communication<br>C) Lack of **trust and awareness** of TCA systems<br>D) **Inadequate infrastructure and resources** for TCA service delivery

For peer review only - http://bmjopen.bmj.com/site/about/guidelines.xhtml

Experiences and meanings of integration

Table 2. Strategies~~Recommendations~~ to increase facilitators and decrease barriers ~~topromote/address~~ Integration, corresponding with study~~responding to~~ findings

Strategies that may enhance TCA integration for essential health services delivery, based on our findings	Strategies that promote FACILITATORS				Strategies that remove BARRIERS			
~~Strategies to promote TCA integration for essential health services delivery, based on our findings~~	Collegiality	Stature	Personal Initiative	Personal Experience	Fragmentation	Isolation	Lack of trust/ awareness	Inadequate infrastructure/ resources
**High level political will required for all strategies**								
Case documentation and sharing across systems, and in the academic literature		✓	✓	✓		✓	✓	
Routine opportunities for interaction and collaboration across systems (eg. health camps, health promotion drives)	✓			✓	✓	✓	✓	✓
Routine opportunities for interaction within co-located facilities (eg. staff meetings)	✓			✓	✓	✓	✓	✓
Rewards for integrative initiative of individuals (eg. challenge grants or institutional recognition)	✓	✓	✓	✓				
Rewards for integrative initiative at systems or facility level (eg. joint~~Joint~~ targets like no. of monthly referrals, no. of cases jointly resolved)		✓		✓	✓	✓	✓	✓
Guidelines for collaboration (criteria and conditions for cross-referral, jointly developed by practitioners, non-clinical aspects of work together, including health promotion and managerial duties)				✓	✓	✓	✓	✓

Merged Cells

Formatted: Font: Font color: Dark Red (×14)

Formatted: Font: Italic, Font color: Dark Red

Formatted: Font: Font color: Dark Red (×3)

Formatted: Indent: Left: 0", Add space between paragraphs of the same style

Formatted: Font: Font color: Dark Red (×7)

Formatted: Font: Italic, Font color: Dark Red

Formatted: Font: Font color: Dark Red (×3)

Formatted: Indent: Left: 0", Add space between paragraphs of the same style

Formatted: Font: Font color: Dark Red (×6)

For peer review only

For peer review only - http://bmjopen.bmj.com/site/about/guidelines.xhtml

References

[1] World Health Organization. WHO congress on traditional medicine 2008: Beijing declaration. WHO,2011.

2 *Resolution of the 62nd World Health Assembly on Traditional Medicine 2009*. EB124.R9. Geneva: WHA; 2009.

3 Bodeker G, Kronenberg F. A public health agenda for traditional, complementary, and alternative medicine. *Am J Public Health* 2002;92:1582–91.

4 Sheikh K, Nambiar D. Government policies for traditional, complementary and alternative medical services in India: from assimilation to integration? *Natl Med J India* 2011;24:245–6.

[5] McLeod H, Chung VCH. The Integration of Traditional and Complementary Medicine in Health Systems. *Presented at the Joint IACA, IAAHS, and PBSS Colloquium.* Hongkong. 2012. http://www.actuaries.org/HongKong2012/Presentations/TBR11_McLeod.pdf (accessed 9 Sep 2013).

[6] Quaiser N. Essentialising the body. Locating medical domination and resistance. *The Newsletter.* Autumn 2013. http://www.iias.nl/the-newsletter/article/essentialising-body-locating-medical-domination-and-resistance (accessed 21 Jan 2013).

[7] Khan S. Systems of medicine and nationalist discourse in India: Towards "new horizons" in medical anthropology and history. *SocSci Med.* 2006;62: 2786-97.

[8] World Health Organization. WHO Traditional Medicine Strategy 2002 – 2005. WHO,2002.

[9] Lohokare M, Davar BV. The Community Role of Indigenous Healers. In: Sheikh K, George A (eds.) *Health Providers in India: On the Frontlines of Change*. New Delhi: Routledge; 2011. p161–81.

For peer review only

[10] Department of Health and Family Welfare. *Mainstreaming AYUSH under NRHM.* http://203.193.146.66/hfw/PDF/ayus.pdf (accessed 31 Mar 2011).

[11] Department of AYUSH. *AYUSH under NRHM.* http://indianmedicine.nic.in/writereaddata/linkimages/4468616884-AYUSH%20under%20NRHM.pdf (accessed 5 Sep 2013).

[12] Unnikrishnan PM, Kumar LHP, Shankar D. Traditional Orthopaedic Practitioners' Place in Contemporary India: A Case Study from Southern India. In: Sheikh K, George A (eds.) *Health Providers in India: On the Frontlines of Change*. New Delhi: Routledge; 2011. p182–99.

[13] Lakshmi JK. Less equal than others? Experiences of AYUSH medical officers in primary health centres in Andhra Pradesh. *Indian J Med Ethics* 2012;9:18–21.

[14] Priya R, Shweta AS. *Status and Role of AYUSH and Local Health Traditions: Under the National Rural Health Mission, Report of a Study*. National Health Systems Resource Centre. 2010.

[15] Barrett S, Fudge C. *Policy and action: essays on the implementation of public policy*. London: Methuen; 1981.

[16] Walt G, Gilson L. Reforming the health sector in developing countries: the central role of policy analysis. *Health Policy Plan* 1994;9:353–70.

[17] Yanow D. *Conducting Interpretive Policy Analysis*. Newbury Park,CA: Sage; 2000.

[18] Ritchie J, Spencer L. Qualitative Data Analysis for Applied Policy Research. In *Analyzing qualitative data*. London: Routledge;1994. p173-94.

For peer review only - http://bmjopen.bmj.com/site/about/guidelines.xhtml

[19] Hollenberg D. Uncharted ground: Patterns of professional interaction among complementary/alternative and biomedical practitioners in integrative health care settings. *Soc Sci Med* 2006;62:731-44.

[20] Broom A, Doron A, Tovey P. The inequalities of medical pluralism: Hierarchies of health, the politics of tradition and the economies of care in Indian oncology. *Soc Sci Med* 2009;69:698–706.

[21] Chung VC, Hillier S, Lau CH, Wong S, Yeoh EK, Griffiths SM. Referral to and attitude towards traditional Chinese medicine amongst western medical doctors in postcolonial Hong Kong. *Soc Sci Med* 2011;*72*:247-55.

[22] Wangchuk P, Wangchuk D, Aagaard-Hansen J. Traditional Bhutanese medicine (gSo-BA Rig-PA): an integrated part of the formal health care services. *Southeast Asian J Trop Med Public Health* 2007;38:161–7.

[23] Bloom H. *The Lucifer Principle. A Scientific Expedition into the Forces of History*. New York: The Atlantic Monthly Press;1995.

[24] Northridge ME, Mack Jr R. Integrating ethnomedicine into public health. *Am J Public Health* 2002;92:1561-61.

For peer review only - http://bmjopen.bmj.com/site/about/guidelines.xhtml

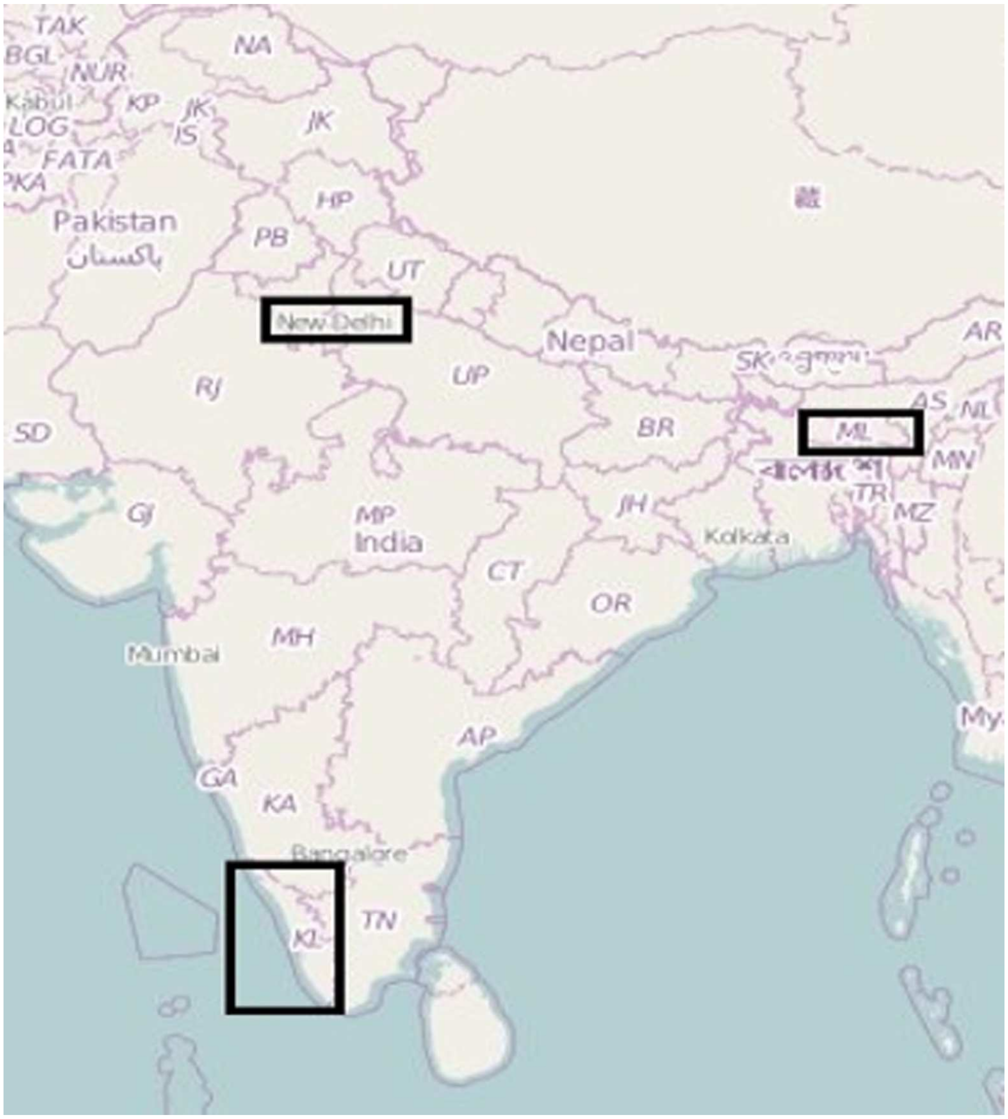

Figure 1. Location of states in India where fieldwork was conducted (New Delhi, Meghalaya (ML), and Kerala (KL))
251x279mm (300 x 300 DPI)
